# Synthesis of protective oral PrEP adherence levels in cisgender women using convergent clinical- and bottom-up modeling

**DOI:** 10.21203/rs.3.rs-2772765/v1

**Published:** 2023-04-21

**Authors:** Lanxin Zhang, Sara Iannuzzi, Ayyappa Chaturvedula, Jessica E. Haberer, Craig W. Hendrix, Max von Kleist

**Affiliations:** 1 Project group 5 “Systems Medicine of Infectious Disease”, Robert Koch Institute, Berlin, Germany; 2 International Max-Planck Research School “Biology and Computation” (IMPRS-BAC), Max-Planck Institute for Molecular Genetics, Berlin, Germany; 3 The University of North Texas; Health Science Center, Fort Worth, United States; 4 Pumas-AI Inc.; 5 Center for Global Health, Massachusetts General Hospital, Boston, United States; 6 Department of Medicine, Harvard Medical School, Boston, United States; 7 Division of Clinical Pharmacology, Johns-Hopkins University, Baltimore, United States; 8 Department of Mathematics and Computer Science, Freie Universität Berlin, Berlin, Germany

**Keywords:** viral dynamics, infection, pre-exposure prophylaxis, HIV, mathematical model, adherence

## Abstract

Globally, most HIV infections occur in heterosexual women in resource-limited settings. In these settings, female self-protection with generic emtricitabine/tenofovir disoproxil fumarate pre-exposure prophylaxis (FTC/TDF-PrEP) may constitute a major pillar of the HIV prevention portfolio. However, clinical trials in women had inconsistent outcomes, sparking uncertainty regarding risk-group specific adherence requirements and causing reluctance in testing and recommending on-demand regimen in women.

We analyzed all FTC/TDF-PrEP trials to establish PrEP efficacy ranges in women. In a ‘bottom-up’ approach, we modeled hypotheses corroborating risk-group specific adherence-efficacy profiles. Finally, we used the clinical efficacy ranges to (in-)validate hypotheses.

We found that different clinical outcomes could solely be explained by the proportion of enrolled participants not taking the product, allowing, for the first time, to unify clinical observations. This analysis showed that 90% protection was achieved, when women took *some* of the product. Using ‘bottom-up’ modelling, we found that hypotheses of putative male/female differences were either irrelevant, or statistically inconsistent with clinical data. Furthermore, our multiscale modelling indicated that 90% protection was achieved if oral FTC/TDF was taken at least twice weekly.

## INTRODUCTION

Human immunodeficiency virus (HIV) infection, the causative agent of the acquired immunodeficiency syndrome (AIDS) [1, 2], constitutes one of the most devastating pandemics to date. While AIDS can be prevented using combination antiretroviral therapy, there is still no cure for HIV infection [3]. Hence, HIV infection necessitates life-long antiviral therapy to prevent AIDS, which can be challenging due to numerous individual and structural barrier [4, 5].

According to UNAIDS, approximately 1.5 million individuals became infected with HIV in 2021 [6] with the sub-Saharan African countries hit hardest. Here, young women are disproportionally affected, accounting for 63% of all new infections in 2021 [7]. Recent estimates in southern Africa state that almost a quarter of the female population in their reproductive age is HIV positive [8]. Consequently, young women in sub-Saharan Africa remain a highly impacted risk group in need of options for HIV prevention. For women, HIV pre-exposure prophylaxis (PrEP) is the only biomedical means to date, by which they have control over HIV self-protection. While novel long-acting PrEP regimen are becoming available [9, 10], PrEP with oral emtricitabine/tenofovir disoproxil fumarate (FTC/TDF) is available as a generic formulation, making it accessible to many resource-limited settings. However, major uncertainties exist regarding adherence requirements in heterosexual women to achieve adequate per-exposure HIV risk reduction. In light of these uncertainties, it is currently recommended that women take FTC/TDF-based PrEP *once daily*; -an adherence requirement that may negatively impact on its uptake and persistence over time in young women.

The *average* PrEP efficacy across an observation cohort in a clinical study is typically quantified as the relative risk reduction to acquire HIV infection in a PrEP cohort versus a control (e.g. placebo) group.

$$\text{average}\;\text{PrEP}\;\text{efficacy}=1-\frac{\text{HIV}\;\text{incidence}\;\text{(intervention)}}{\text{HIV}\;\text{incidence}\;\text{(control)}},$$

where the HIV incidence is computed by dividing the number of infections in the respective arm, by the observation horizon (e.g., 5 infections in 2780 person-years). Because HIV transmission per *sexual* exposure is relatively low [11], only a few transmission events are typically observed in the control group, even in very large clinical trials with substantial longitudinal follow-up [12]. Consequently, the vast majority of clinical trial participants do not contribute to ‘measurements’ that can be used to quantify PrEP efficacy [13]. This circumstance has important consequences with regards to interpreting clinical data on PrEP efficacy: (i) Firstly, clinical estimates *average* over possibly very heterogeneous risk-, as well as PrEP-adherence behavior. (ii) Secondly, clinical estimates of *average* PrEP efficacy are *statistically* uncertain, due to the small number of ‘measurements’ in combination with aforementioned heterogeneity. (iii) Thirdly, because of the statistical uncertainty and the requirement for large and long-lasting clinical studies, the possibility to clinically identify and verify causative factors that influence *per-exposure* HIV risk reduction is greatly limited.

In heterosexual women the range of clinically estimated *average* PrEP efficacy is particularly vast [14, 15]. For example, the Partners-PrEP study estimated a 66–71% efficacy of TDF +/− FTC in women overall, which increased to ~90% in individuals with detectable drug levels [16]. The TDF2 study reported an average efficacy of 49% [17] and a clear association with drug adherence. HPTN 084 [9] reported an HIV incidence of 1.85 per 100 person-years in the FTC/TDF control arm at an *average* adherence of about 42%, with ≤4 out 36 infected individuals showing evidence for *some* PrEP adherence around the time of infection [18]. Fem-PrEP [19] and VOICE [20] reported no incidence reduction for FTC/TDF-based PrEP in women. In Fem-PrEP about 15% of infected individuals had detectable drug levels; in VOICE, adherence was low (~30%) and intermittent, such that it is unclear whether the product was used at the time of HIV exposure.

This apparent inconsistency of clinical outcomes may have sparked uncertainty regarding adherence requirements and caused reluctance in testing and recommending on-demand regimen in women.

For FTC/TDF-based oral PrEP, some early studies pointed towards lower *average* risk reduction in heterosexual women, compared to men-who-have-sex-with-men (MSM) [14, 19, 20]. However, it is unclear to date, whether this putatively lower efficacy is a consequence of intrinsic differences in physiology and pharmacokinetics at the site of exposure, or whether it is an artefact of poorly quantified and differing levels of adherence across studies, as many infected participants in these trials were simply not taking PrEP around the time of HIV exposure.

For developing PrEP guidelines, the existence of intrinsic differences would be relevant, as it necessitates specific recommendations on, for example, minimal adherence levels, which can also affect the uptake and persistence of PrEP in the considered risk group [21, 22]. Current WHO guidelines for PrEP differentiate between heterosexual women and MSM [23]. While PrEP on demand is considered safe in MSM based on the IPERGAY and PREVENIR studies [24], no such study has been attempted in women; therefore, on-demand PrEP is not recommended for heterosexual women. The reluctance to test on-demand PrEP in women is partially originating in prior mathematical modelling studies which suggest higher adherence in women vs. MSM, for the same level of protection [25]. Notably, these modelling studies were partially motivated by results from the Fem-PrEP [19] and VOICE [20] study that failed to show efficacy in women, but also showed the lowest adherence levels of all investigated studies (hence may be uninformative regarding the adherence-efficacy profile).

**Figure 1:**
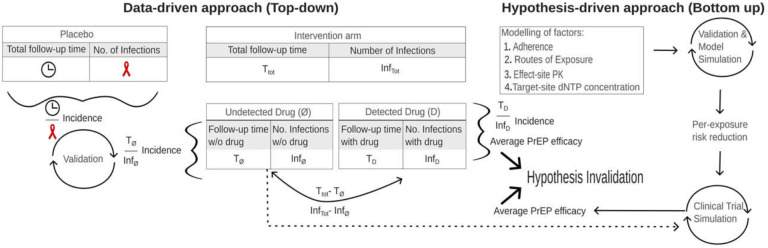
General approach of combining data-driven ‘top-down’ and hypotheses-driven ‘bottom-up’ modelling to investigate FTC/TDF-based PrEP efficacy in women. In the ‘top-down’ approach, we solely used clinical data to infer PrEP efficacy in women with detectable plasma TFV (women who took *some* product). Based on pharmacokinetic models, we could dichotomize PrEP interventions arms. When drug was undetectable, incidences corresponded to placebo incidences, thus efficacy was assumed to be 0%. By simulating this placebo-like sub-cohort of the PrEP intervention arm, we could estimate drug efficacy in individuals with detectable drug. In the ‘bottom-up’ approach we implemented all previously proposed hypotheses (exposure, drug potency, drug pharmacokinetics) that aim at mechanistically explaining distinct efficacy and adherence-efficacy requirements in women (in comparison to men-who-have-sex-with-men; MSM) using advanced multi-scale modelling and simulation. In a final step, we assessed whether proposed hypotheses hold up against clinically observed outcomes.

In this work, we used two independent approaches to quantify the population-specific adherence-protection relationship for PrEP in women (illustrated in Fig. 1): In a data-driven ‘top-down’ approach, we solely utilized clinical data to quantify PrEP efficacy. In analogy to the analysis of early PrEP studies in MSM [26], we used population pharmacokinetic models, to dichotomize the intervention arms into sub-cohorts of product non-taking vs. product taking. Simulation of these sub-cohorts allowed us to estimate PrEP efficacy and corresponding confidence bounds for each clinical study in individuals who took *some* of the products.

In an independent, hypotheses-driven, ‘bottom-up’ approach, we tested mechanisms that have been proposed to explain risk group-specific adherence-protection relationships, such as the types of exposure (receptive vaginal vs. anal intercourse), potentially distinct drug potency, as well as drug concentrations at these exposure sites. These mechanisms were integrated into an advanced multi-scale modelling framework that has been consecutively developed over the past 10 years [27–33]. This model allowed us to synthesize adherence-protection profiles for each proposed hypothesis and all their combinations.

Finally, mechanistic predictions were evaluated in the light of the clinical data (‘top-down’ approach). Most importantly, this final model verification approach allowed us to statistically rule in/out proposed mechanisms on differential PrEP efficacy in heterosexual women and inform minimal adherence requirements in this risk group.

## RESULTS

### Pharmacokinetic Modelling.

To fully reflect the pharmacokinetics (PK) of FTC/TDF, we utilized the models by Burns et al. and Garrett et al. [34, 35], which were trained on rich pharmacokinetic data from the MTN-001, as well as the NCT010330199 and NCT02357602 clinical trials (details in the Methods section). Importantly, these models consider relevant inter-individual PK differences and establish the link between FTC/TDF dosing, prodrug (FTC and tenofovir; TFV) concentration-time profiles in the blood plasma, as well as the pharmacologically active metabolites tenofovir diphosphate (TFV-DP) and emtricitabine triphosphate (FTC-TP) in peripheral blood mononuclear cells (PBMCs). An example of model-simulated concentration-time profiles for a seven-day course of oral 200/300mg FTC/TDF in a cohort of 1000 virtual individuals is shown in Suppl. Fig. S1.

Based on these models, we first established the link between dosing frequency and detectable plasma TFV (> 0.001μM), which was the adherence marker used in Partners-PrEP, TDF2, VOICE and HPTN084. Notably, if a sample is taken from a person, who takes TDF or FTC/TDF once a week, this sample will have detectable plasma TFV was above the limit of detection of 0.001μM with 57% probability (IQR: 46–71%), Supplementary Fig. S2. For a person taking 2 pills a week on average, the probability is already 88%, and 96% for 3 pills a week, Supplementary Fig. S2. In other words, individuals without detectable plasma TFV levels have taken TDF or FTC/TDF less than once weekly, if at all. For Fem-PrEP the plasma TFV threshold concentration for assessing adherence was <0.035μM, which corresponds to having taken FTC/TDF (respectively TDF) less than twice per week, if at all (Suppl. Fig. S3).

#### Data-driven ‘top-down’ analysis of PrEP trials in women.

**Figure 2:**
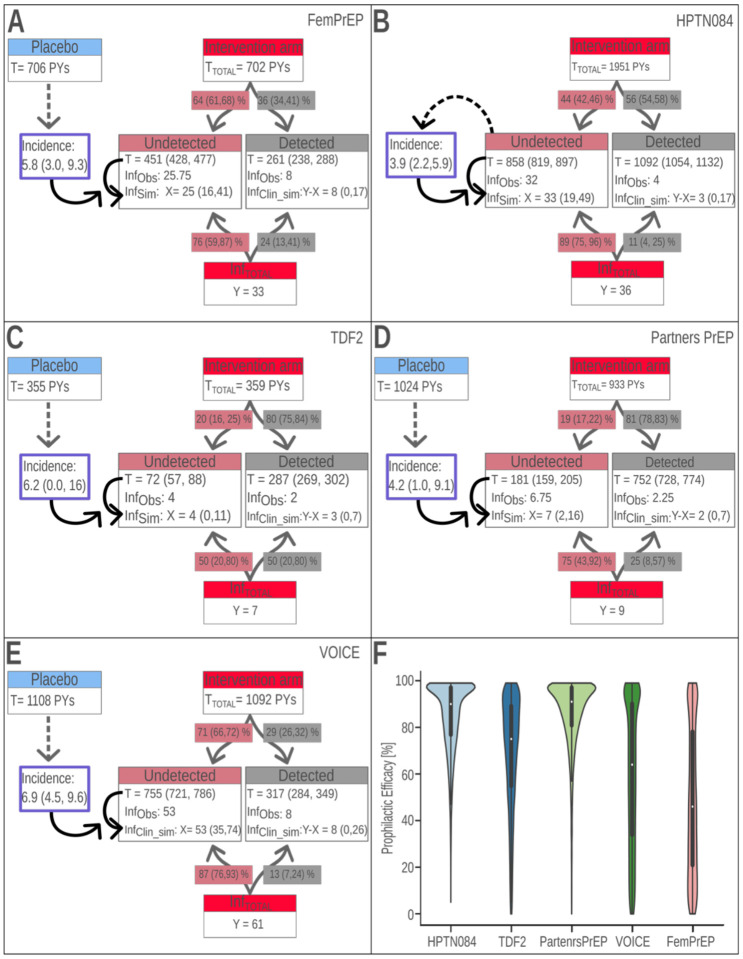
Summary of clinical trials evaluating FTC/TDF-based PrEP in women. **A-E**: Summary of clinical trials: The total observation time (T_Total_ in person-years) in the respective trials was dichotomized into “detected” and “undetected” based on the fraction of measurements with detectable plasma TFV. Likewise, the total number of infections ‘Y’ were proportionally assigned based on the fraction of infected individuals that had detectable plasma TFV. We then stochastically simulated the “no-drug intervention” (Methods and Supplementary Text S1) to compute the number of infections in the “no-drug intervention” arm ‘X’. From both the total observed number of infections ‘Y’ and the simulated number of infections in the “no-drug intervention” arm, ‘X’, we could compute the number of infected individuals with detectable plasma TFV as Y - X. **F**: The violin plots indicate the probability distributions of PrEP efficacy *P*_*drug*_(*φ*) when conducting clinical trial simulation with only data provided in the respective studies (Supplementary Text S1). The width indicates the likelihood of a particular efficacy. Square-shaped violin plots indicate uninformative clinical trials (Fem-PrEP, VOICE), i.e. no conclusion can be drawn with regards to the PrEP efficacy, whereas sharply concentrated distributions (HPTN084, Partners-PrEP) are informative regarding PrEP efficacy.

Next, we simulated PrEP trials that assessed the impact of FTC/TDF-based PrEP on HIV incidence (Methods). We first evaluated corresponding incidence rates in placebo arms, as reported for Partners-PrEP, TDF2, Fem-PrEP and VOICE, Table 1. We then evaluated the intervention arms: Based on the relation between adherence and detectable TFV in blood plasma (Suppl. Fig. S2–3), we assumed that individuals with undetectable drug may not have taken FTC/TDF at all. For those individuals we assumed a PrEP efficacy of 0%. The proportion of random samples with undetectable plasma TFV in the FTC/TDF intervention arm was 19% in Partners-PrEP and TDF2 study, 44% in HPTN084, 64% in Fem-PrEP and 71% in the VOICE (Fig. 2A–E). We then simulated the intervention arms of these studies by assigning a fraction of observation period (person-years) to a “drug undetected” sub-cohort, as well as by assigning a fraction of the infected individuals to the “drug undetected” sub-cohort, if TFV was not detectable (Fig. 2A–E, Table 1). The remaining observation time and number of infected individuals was assigned to the sub-cohort of the intervention arm where individuals had detectable drug.

### Validity of dichotomization into “detectable” vs. “undetectable” drug.

To assess our assumption of 0% efficacy in the “drug undetected” intervention sub-cohort, we first computed incidences in this sub-cohort directly from the respective studies (Fig. 2A–E). These incidences corresponded well with the incidences in the respective placebo arms, albeit slightly (but insignificantly) higher, indicating that we may safely assume that the efficacy of FTC/TDF in non-adherent individuals (“drug undetected” intervention sub-arm) was 0%. We then simulated the “drug undetected” sub-cohort, as explained in the Methods section, by taking both the follow-up time, as well as “drug undetected” incidences from the respective studies as input (Methods and Supplementary Text S1). Our clinical trial simulation took two sources of variability into account: (i) uncertainty in the incidence rate, as well as (ii) intrinsic stochasticity. While the former is standard procedure, the latter is often ignored, but relevant here because infection is an extremely rare event in the investigated clinical trials, contributing to intrinsic randomness. The resultant number of infections (and their uncertainty) are depicted in Fig. 2A–E and are highly consistent with the clinically reported numbers. As a further validity check, we also depicted the simulation-derived incidence rates in Suppl. Fig. S4 together with data-derived placebo and “drug undetected” incidences. Notably, because our simulation took both, intrinsic stochasticity as well as uncertainty in the actual incidence rate into account (Supplementary Text S1), and therefore corresponding confidence intervals are wider than those derived from the clinical data alone (which neglect ‘intrinsic stochasticity’), Suppl. Fig. S4.

### PrEP-efficacy in individuals with detectable drug.

Next, we calculated the range of PrEP efficacies in individuals with detectable drug (individuals who took *some* product). For this, we only used reported data, as well as the simulated “drug undetected” sub-cohorts (previous paragraph). For each simulation, we calculated the number of infections (and its uncertainty) in the subset of individuals with “detectable drug” by subtracting the number of infections derived from the “drug undetected” simulation from the total number of infections reported in the respective clinical study. Since we perform stochastic simulations, this yields a probability distribution. I.e. we derive the probability to observe *x* = 0, …, *N* infected individuals in the “drug detected” sub-cohort *P*_drug_(*x*); details in Suppl. Text S1. Using the incidences from the “drug undetected” sub-cohort and the entire range of theoretically possible PrEP efficacies (incidence reductions) *φ*, we then calculated the probability of. *φ*, conditioned on observing *x* infections in a simulated trial *P*_drug_(*φ*|*x*). Combining both, we could deduce information about PrEP efficacy in individuals with detectable drug $P_{\text{drug}}(\varphi )=\sum_{x}P_{\text{drug}}(\varphi |x)\cdot P_{\text{drug}}(x)$, as shown in Fig.2F. These analyses give two important insights: (i) The VOICE and Fem-PrEP studies span almost the entire theoretically possible range of PrEP efficacy in individuals taking (some of) the product. This essentially means that these studies are uninformative with regards to PrEP efficacy in individuals taking *some of* the product, as indicated by the almost uniform distributions in Fig. 2F. (ii) The TDF2 study is also relatively uninformative with regards to inferring PrEP efficacy in individuals with detectable drug, but rather points towards higher efficacy. (iii) The remaining studies (HPTN084 and Partners-PrEP) point towards high PrEP efficacy in women taking *some of* the product. I.e. the vast majority of *P*_drug_(*φ*) lies above 80% and the median efficacy is ~90%.

Importantly, this analysis did not yet make any assumption about adherence in individuals with detectable plasma TFV, other than that individuals with undetectable drug have 0% PrEP efficacy; i.e. similar to placebo (Supplementary Fig. S4).

#### Mechanism-driven ‘bottom-up’ modelling of PrEP efficacy.

**Figure 3:**
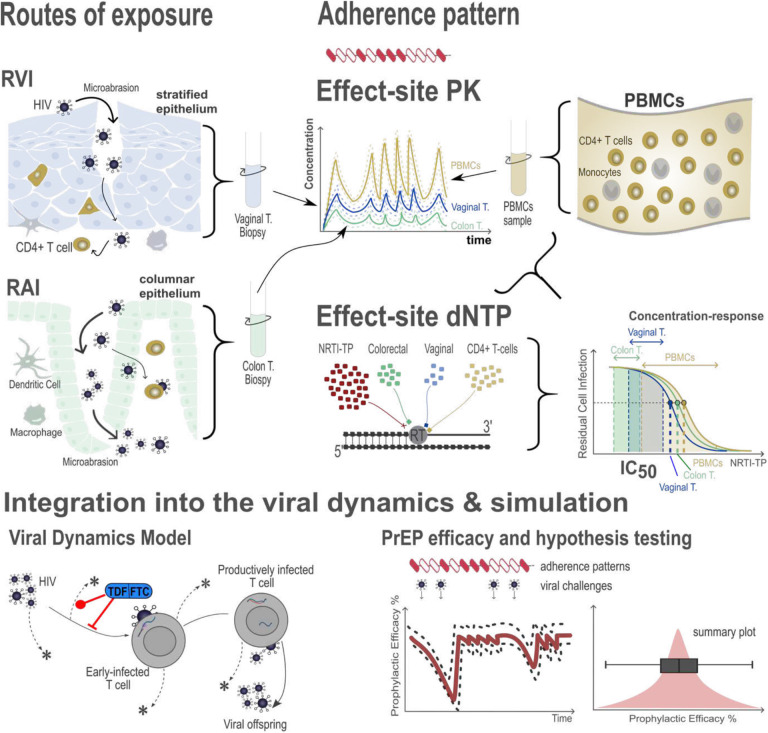
**Bottom-up approach** for testing the concomitant effects of proposed mechanisms (pharmacokinetics, drug potency, exposure) on PrEP efficacy in heterosexual women: Exposure may either occur via receptive vaginal- or anal intercourse (RVI or RAI). Effect-site drug concentrations time-courses were either related to local tissue biopsies or PBMCs, and via pharmacokinetic models related to adherence patterns in individuals taking PrEP. Concentration-response profiles and drug potency were then computed based on deoxynucleoside triphosphate (dNTP) concentrations in local-tissue or CD4+cells and integrated with the effect-site pharmacokinetics to estimate the time-course of drug inhibition of viral replication shortly after exposure. By integrating the viral dynamics with adherence, effect-site pharmacokinetics and drug inhibition, we computed the temporal profile of prophylactic efficacy, i.e. the reduction of infection incidence if virus exposure happened at some time *t*. By integrating over all possible times *t*, we derive a summary statistic for an individual with a given adherence and pharmacokinetic profile and an unknown virus exposure time. Final PreP efficacy estimates summarize predictions over many adherence profiles and 1000 virtual patients (= pharmacokinetic parameter sets).

Next, we assessed proposed mechanisms corroborating risk-group specific adherence-efficacy profiles in women. Because it is almost impossible to conduct clinical trials that systematically test the influence of *the exposure route, exposure site pharmacokinetics*, *and -drug potency*, we used integrative (‘bottom-up’) mathematical modeling of available *in vitro* and *ex vivo* data to study the potential effects of aforementioned factors on PrEP efficacy (Supplementary Texts S2–3). This approach takes models of e.g. (a) *per-exposure* risk of infection, (b) plasma, intracellular and exposure-site pharmacokinetics, (c) the molecular mechanisms of action (MMOA) of considered drugs and (d) their pharmacological interaction into account and combines them into a single framework, allowing us to study the concomitant effects of aforementioned factors, Fig. 3: Our implemented population pharmacokinetic models [34, 35] (details in the *Method* section) establish the link between individual adherence patterns and target-site drug concentrations to assess the impact of *exposure-site pharmacokinetics*. Models of the molecular mechanisms of action of the considered drugs allow us to integrate the combined antiviral effect of FTC and TDF, as well as to study the effects of differential deoxynucleoside triphosphate (dNTP) levels on the *exposure-site potency* of FTC/TDF. Models of virus exposure allow investigating the role of the *route of HIV exposure*, either via receptive anal-(RAI), or receptive vaginal intercourse (RVI). All of these mechanisms can be integrated to determine the initial viral dynamics, from which we can compute PrEP efficacy (reduction in infection risk) and adherence-efficacy profiles using the methods developed in [33].

Notably, each of these ‘building blocks’ has been individually verified or developed based on available *clinical* and *in vitro* data [36–42], reflecting the current state-of-knowledge of the mechanisms of action of FTC/TDF-based PrEP at the multiple scales that determine PrEP efficacy.

**Figure 4:**
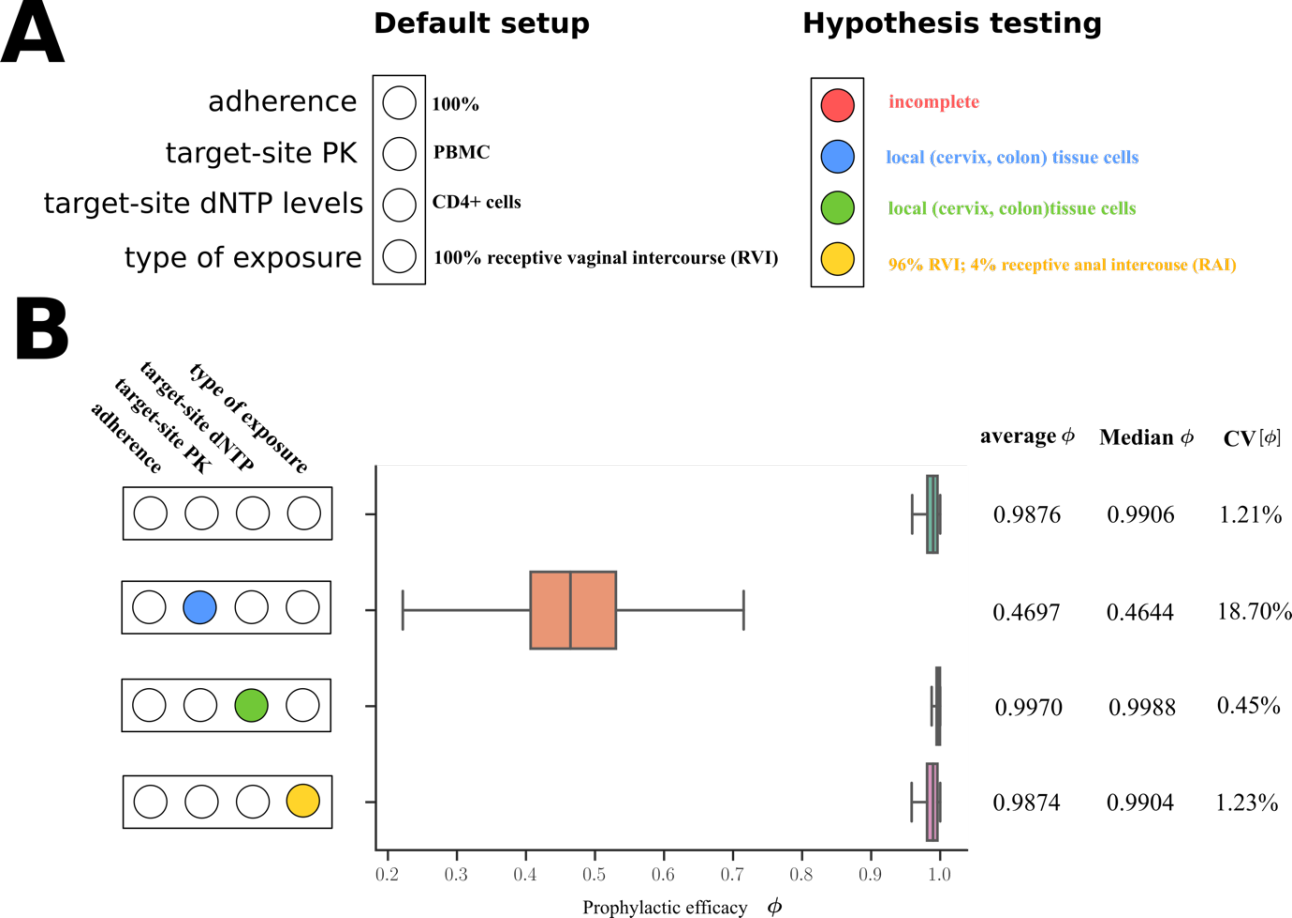
Hypothesis testing and impact of single mechanisms on PrEP efficacy. **A**: ‘Traffic-light’ based set-up to simulate the impact of hypotheses in isolation or in combination: The first ‘traffic-light’ indicates whether adherence is complete (baseline scenario; light ‘off’) or incomplete (red light ‘on’), the second light indicates whether intracellular tri-phosphate drug concentrations in peripheral blood mononuclear cells (PBMCs) were used as a marker for the target compartment (baseline scenario; blue light ‘off’), or whether local colorectal (RAI) and vaginal/cervical (RVI) cellular drug concentrations (‘blue light on’) were used. The third light indicates whether endogenous deoxynucleotide (dNTP) levels corresponding to CD4+ T-cells (baseline scenario, ‘green light off’) or those corresponding to colo-rectal and vaginal/cervical cells (‘green light on’) were used. The last ‘traffic light’ highlights whether only receptive vaginal intercourse (RVI, baseline scenario) was simulated or whether 4% of exposures occurred via the receptive anal route (‘yellow light on’). **B**: Population prophylactic efficacy estimates considering different hypotheses in isolation. N = 1000 virtual patients were sampled. 200 mg FTC and 300 mg TDF was ingested with an adherence level of 100%. The effect of pharmacokinetics at exposure sites, endogeneous dNTP level and routes of exposure (RAI and RVI) was investigated respectively. The whisker of the box plot represents 1.5 × inter-quartile range (IQR) and CV denotes the coefficient of variation of the average prophylactic efficacy.

For evaluation, we consider a combinatorial approach to test the proposed mechanisms alone and in combination. We will designate the respective simulation setting in analogy to a light switch, Fig. 4A, where each of the ‘four lights’ corresponds to a mechanism that we model:

1. *Adherence*. We either perform simulations where the adherence is complete (baseline scenario; light ‘off’) or incomplete (red light ‘on’). The goal is to assess the maximally achievable protection (complete adherence), as well as to evaluate minimum requirements for adherence.
2. *Exposure-site pharmacokinetics*. TFV and FTC do not directly exert antiviral activity. Rather, the drugs need to be taken up by HIV target cells and be intracellularly converted into their active moieties, tenofovir diphosphate (TFV-DP) and emtricitabine triphosphate (FTC-TP). Unfortunately, the kinetics of TDF and TFV in blood plasma poorly correlate with the kinetics of the intracellular moieties and pharmacological response [43]. It is therefore required to model the kinetics of intracellular TFV-DP and FTC-TP in order to assess the efficacy of these drugs.
  To quantitatively assess the impact of local intracellular drug concentrations in the present study, we either used intracellular tri-phosphate drug concentrations (FTC-TP and TFV-DP) in systemic peripheral blood mononuclear cells (PBMCs), which contain high proportions of HIV target cells (CD4+ T-cells) as a marker for the target compartment (blue light ‘off’), or we used drug concentrations in colo-rectal (RAI) and vaginal (RVI) tissue homogenates (‘blue light on’), which contain low CD4+ T-cells contents, but are derived from the virus exposure site; Details in Supplementary Text S3.
3. *Exposure-site drug potency*. TFV-DP and FTC-TP are both competitive inhibitors of HIV reverse transcription [18]. The potency of competitive inhibitors can thus be altered by endogenous substrate concentrations that these drugs compete with [19, 20]. Cottrell et al. [25], hypothesized that different concentrations of dNTP at the colorectal vs. the vaginal exposure site create differences in efficacy and adherence requirements for MSM vs. heterosexual women. To assess whether the drugs have a distinct drug potency at the vaginal and colo-rectal exposure site, we utilized local tissue dNTP measurements in a mechanistic model of the molecular mode of action of TFV-DP and FTC-TP [31] (‘green light on’), or, in a baseline scenario, used dNTP concentrations in CD4+ T-cells [44] (‘green light off’).
4. *Exposure route*. Heterosexual women may be exposed via RAI, or RVI, where exposure route may involve different levels of HIV risk reduction and different minimal adherence requirements. We modelled exposure either purely via RVI (baseline scenario; ‘yellow light off’) or we included 4% anal exposures (‘yellow light on’, details provided in the Methods section, as well as Supplementary Text S2).

### PrEP efficacy in the baseline scenario.

**Figure 5:**
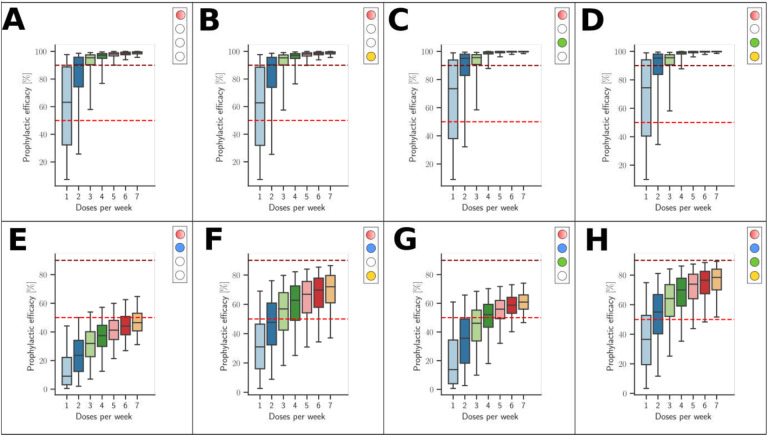
Impact of combined mechanisms on PrEP efficacy and adherence requirements for different hypotheses. **A-H**: Model-predicted prophylactic efficacy if FTC/TDF was taken one-, two-, …, seven days per week on average. Boxplots show median efficacy and IQR and whiskers extend to 2.5%-97.5% range. 90- and 50% efficacy are highlighted for visual guidance using horizontal dashed black and red lines respectively. **A**: Baseline scenario with different levels of drug adherence. **B**: Mixed vaginal- and anal exposure. **C**: Altered drug potency at site of exposure (through dNTP levels). **D**: Mixed exposure and altered drug potency. **E**: Drug concentration in local tissue homogenates are used as an effect compartment marker. **F**: Concentrations in local tissue homogenates are used and mixed exposures occur. **G**: Concentrations in local tissue homogenates and altered drug potency are used. **H**: Concentrations in local tissue homogenates, altered drug potency and mixed (anal and vaginal) exposures are simulated.

We simulated tenofovir and emtricitabine pharmacokinetics following daily intake of FTC/TDF in 1000 virtual individuals based on previously implemented population-pharmacokinetic models in women [34, 35]. Plasma FTC and TFV concentrations reach their steady state after approximately one and three dosing events respectively (Supplementary Fig. S1A–B). Intracellular (PBMC) FTC-TP and TFV-DP concentrations reach their respective steady state after one and seven dosing events respectively (Supplementary Fig. S1C–D). As a reference, we depict target concentrations that would prevent 50- and 90% infections respectively if the drugs were used in isolation and if endogenous dNTP levels at the target-site coincide with dNTP levels measured in CD4+ T-cells [44]. Using this default setting, i.e, if PBMCs were a marker of PrEP efficacy and individuals were 100% adherent, emtricitabine-mediated protection would be larger than tenofovir-mediated HIV protection: At steady state, FTC-TP concentrations would fluctuate around the 90% preventive concentration whereas TFV-DP would reach concentrations that yield between 50 to 90% protection in the majority of individuals (Supplementary Fig. S1C–D). By mechanistically modeling the intracellular synergistic interaction between tenofovir and emtricitabine [31], we calculated the instantaneous efficacy of the drug combination (Supplementary Fig. S4) and based thereon the prophylactic efficacy of FTC/TDF (Fig. 4B) for the baseline scenario (individuals are 100% adherent, PBMC concentrations are a marker of efficacy, dNTP concentrations from CD4+ T-cells and 100% receptive vaginal exposure). This scenario predicted high *average* prophylactic efficacy (98%) in fully adherent women after receptive vaginal intercourse (RVI). Simulation results for periodic, but incomplete adherence are depicted in Fig. 5A and show that if FTC/TDF was taken with an adherence of 14% (once weekly) median efficacy was 65% (IQR: 35–90%), while with two and three doses per week adherence median efficacy climbs to 90% (IQR: 75–96%) and 96% (IQR: 90–98%).

Before challenging all models with reported and simulated clinical trial outcomes, however, we will first assess which of the aforementioned hypotheses – alone or in combination – alter the predicted PrEP efficacy in the combined model; I.e.: Which are *relevant* for determining PrEP efficacy?

### Impact of individual mechanisms on PrEP efficacy.

In order to study the importance of aforementioned mechanisms, we next evaluated the individual effects of discussed mechanisms on PrEP efficacy, if individuals fully adhered to the daily FTC/TDF regimen. This allows us to ask: How efficient can PrEP be in a best-case scenario, when considering aforementioned mechanisms in cisgender women?

### Receptive anal intercourse (RAI):

We estimated the proportion of RAI events and the infection probabilities associated with each mode (RVI vs. RAI) of exposure for heterosexual intercourse from average infection probabilities for heterosexual intercourse [11], as well as the fraction of RAI attributable infections (Methods section and Supplementary Text S2). We computed a proportion of 4% RAI events on average for heterosexual female exposure could resemble a realistic cohort. The higher transmissibility of HIV associated with RAI, may *on average*, be associated with larger numbers of viruses breaching physiological barriers, since HIV target cells in the colon are shielded by only a single-cell epithelial layer, unlike the multi-layer epithelial structure at vaginal exposure site, Fig. 3. Hence larger inoculum sizes may be encountered during receptive anal intercourse, as implemented in our modelling approach, Supplementary Text S2.

Notably, when simulating heterosexual exposure with 4% RAI, the overall prophylactic efficacy of once-daily FTC/TDF in fully adherent individuals did not markedly change in comparison to the baseline scenario (only RVI; Fig. 4B).

### Exposure-site drug potency (local dNTP concentrations).

Both, emtricitabine triphosphate (FTC-TP) and tenofovir diphosphate (TFV-DP) compete with their endogenous nucleotide counterparts (dCTP and dATP respectively) and hence, concentrations of these endogenous nucleotides may alter their potency. Cottrell et al. [25] had previously reported local concentration ratios of FTC-TP to dCTP and TFV-DP to dATP in vaginal-, cervical- and colonic tissue. Consequently, we evaluated if the reported dNTP concentrations would change the potency of the drugs and their prophylactic efficacy. Based on our previously developed [32] and validated [46] molecular mechanisms of action model, we could use reported concentrations ratios to assess whether the potency of the drugs was altered in vaginal cervical and colon tissue, Suppl. Text S3.

These evaluations indicated that the potency of TFV-DP in the colon would be identical to PBMCs (IC_50_ ~0.1μM), marginally greater in cervical tissue (IC_50_ ~0.05μM) and slightly lower in vaginal tissue cells (IC_50_ ~0.15μM). The potency of FTC-TP would marginally increase (IC_50_ = 0.39–0.49μM) in all three tissues, compared to the PBMCs IC_50_ = 0.85μM, which denotes the effect compartment marker in the baseline simulation scenario. Consequently, the prophylactic efficacy would be marginally increased (99%) in comparison to the baseline scenario (98%), if local dNTP concentrations were altered according to Cottrell’s reports [25], Fig. 4B.

### Drug concentrations in exposed tissue.

In the baseline modeling scenario, we used drug concentrations in PBMCs as a marker of the relevant concentrations at the site of exposure. The reason is that PBMCs contain a large fraction of HIV target cells (CD4+ T-cells) [47]. Both FTV and FTC are actively taken up by cells and converted by intracellular kinases into their active metabolites (FTC-TP and TFV-DP) [43]. This essentially implies that concentrations of both FTC-TP and TFV-DP may be different in different cell types. However, concentrations in local tissue- or cell homogenates from virus exposure sites are often used as effect site markers. Notably, these mixtures may contain very small proportions of the relevant CD4+ T-cells.

We next investigated the relationship between TFV-DP and FTC-TP concentrations in PBMC vs. local tissue- or cell homogenates. For hypothesis testing, we additionally predicted prophylactic efficacy under the assumption that TFV-DP/FTC-TP concentrations in local tissue homogenates coincided with effect-site concentrations.

We identified 3 studies with 8 dosing regimens for FTC-TP and 5 studies reporting 10 dosing regimens for TFV-DP that report local TFV-DP or FTC-TP concentrations [36–42]. By simulating the respective dosing regimens using our pharmacokinetic models (Methods section), we enabled a direct comparison of measurements between studies and vs. our pharmacokinetic model (Supplementary Text S3). In brief, with regards to drug measurements in PBMCs, the studies showed remarkable consistency between each other and with our model predictions (Supplementary Text S3). We then calculated local:PBMC drug concentration ratios, which again yielded remarkably consistent results between the different studies and across the distinct dosing regimen Supplementary Text S3. Since the local:PBMC drug concentration ratios were consistent across different dosing schedules and measurement time-points, it was appropriate to assume that local drug kinetics were proportional to drug kinetics in PBMCs. Based on these analyses we computed *weighted geometrical means* of the local:PBMC drug concentration ratios. This analysis indicated that TFV-DP concentrations were about 2-fold higher in colon homogenates, compared to PBMC and 16-fold lower in vaginal tissue. FTC-TP concentrations in colon tissue were lower compared to PBMC. FTC-TP vaginal tissue homogenate concentrations were about 16-fold lower than in PBMC (precise numerical values in Supplementary Text S3).

To evaluate the hypothesis that local tissue concentrations represent a marker of efficacy, we subsequently used the derived local tissue:PBMC concentration ratios to predict local tissue pharmacokinetics. Using this data, model-predicted prophylactic efficacy was markedly reduced. I.e., best-case PrEP efficacy in fully adherent individuals was only 47% (IQR: 42–55%), compared to the baseline scenario (efficacy: ~98%).

In summary, our simulations point out that the type of exposure, as well as local dNTP concentrations have little impact on PrEP efficacy when considered in isolation. To the contrary, if tissue homogenates were a marker for the relevant effect-site concentrations, PrEP efficacy was markedly reduced, even in fully adherent individuals. Next, we assess whether the hypothesized mechanisms when considered in combination, impact PrEP efficacy differently, and whether they set certain minimum requirements for PrEP adherence to protect women from acquiring HIV infection.

### Combined impact of hypothesized mechanisms on PrEP efficacy and adherence requirements.

We simulated all combinations of aforementioned hypothesis and assessed the impact of incomplete adherence on HIV protection, Fig. 5. In the baseline scenario (Fig. 5A), prophylactic efficacy was high (median > 95%) when FTC/TDF is taken at least three times per week, on average. Efficacy starts to drop sharply when FTC/TDF was taken once a week, but the median efficacy is still about 65% (IQR: 35–90). Similar results are obtained when mixed (RVI, RAI) exposures occur, when local dNTP levels were altered, or when the two mechanisms co-occur (Fig. 5A–D). To the contrary, when drug levels in tissue homogenates were considered, drug efficacy dropped considerably, with median efficacy being <50% in fully adherent individuals (compare also Fig. 4), dropping gradually to <10% in individuals that take FTC/TDF once per week on average, Fig. 5E. When both drug and dNTP levels from tissue homogenates were considered (Fig. 5G), the efficacy-adherence profile is elevated by about 10% compared to Fig.5E. Considering mixed (vaginal and anal) exposures, drug concentrations in homogenates with/and without altered dNTP concentrations yield similar efficacy-adherence profiles (Fig. 5F,H): Median achievable prophylactic efficacy in fully adherent women is below 80% and gradually decreases to ~50% in women that take FTC/TDF two days a week on average and ~30% for women who take FTC/TDF one day per week on average.

In summary, if drug concentrations in local homogenates are the relevant marker for the prophylactic efficacy, then FTC/TDF would incompletely protect women from HIV infection, even in fully adherent individuals. In the same simulations, protection from vaginal exposure would be lower (Fig. 5E) than protection from anal- and mixed exposure (Fig.5F). This difference is explained mainly because vaginal tissue homogenates indicate depletion of both drugs in comparison to PBMCs, whereas colon homogenates suggest an enrichment of TFV-DP and a depletion of FTC-TP (Supplementary Text S3). In all scenarios where local tissue concentrations are considered, incomplete adherence has a gradual effect on prophylactic efficacy. To the contrary, if drug concentrations in PBMCs were the relevant concentration marker for PrEP, HIV protection would be high, as long as individuals take PrEP three times a week, or more often (Fig. 5A–D).

Next, we will evaluate which predicted adherence-efficacy profiles, and thus which of the tested mechanisms are consistent with outcomes of clinical trials in women.

For this, we simulate the clinical trials as before, but scale the trial-specific incidences with the computed prophylactic efficacies from Fig. 5A–F. This allows us to compare our simulations directly with reported clinical outcomes and statistically challenge all tested hypotheses with clinical data.

#### Challenging ‘bottom-up’ modelling of PrEP efficacy with clinical data.

In the following, we tested whether ‘bottom-up’ inferred PrEP efficacies (Fig. 5) resulted in infection numbers that are inconsistent with clinical data. This allowed to rule out mechanistic hypotheses that were postulated to explain efficacy and risk group-specific adherence requirements in women.

### Individuals with detectable drug.

We simulated the sub-cohort of the intervention arm, where individuals had detectable drug, by sampling from the “drug undetectable” incidence (Suppl. Text S1) and multiplying with the weighted average PrEP-efficacies depicted in Fig. 5 (adherence scheme consistent with the TFV detection dichotomy used in clinical trial analysis, Suppl. Fig. S6).

A head-to-head comparison between clinical trial results (utilizing the predicted uncertainty in the number of infections) and predicted number of infected individuals in the intervention arms, with- and without detectable drug concentrations is shown in Table 2.

The TDF2, Fem-PrEP and VOICE studies do not allow us to distinguish between any of the hypotheses (Table 2) as already suggested by Fig. 2F. For Fem-PrEP and VOICE only 29–36% of the intervention arm did take the study drugs, overall contributing too little data to quantify prophylactic efficacy. For TDF2, the observation time was too short.

By comparing our simulations with the remaining clinical trials (Partners-PrEP and HPTN084; Table 2), we observed that any simulation scenario, in which drug concentrations in PBMCs were used as a marker of efficacy (Fig. 5A–D), was generally in agreement with reported clinical outcomes. To the contrary, if local (vaginal, colorectal) drug concentrations were considered as a marker for the prophylactic efficacy, corresponding clinical trial simulations were either statistically incongruent with clinical data (*P* < 0.05), or are statistically unlikely (*P* < 0.1), (Table 2, Fig 5.E-H). This finding strongly argues that drug concentrations in PBMCs, and not local tissue concentrations, are a more appropriate marker for determining PrEP efficacy in women. Moreover, from Fig. 4 and Fig. 5A–D, we can conclude that none of the other hypotheses (local dNTP concentrations, frequency of receptive anal intercourse) *profoundly* alters prophylactic efficacy, except that including local dNTP levels slightly upwards shift the adherence-efficacy profiles when local tissue concentrations are considered.

All in all, our simulations highlight that the VOICE and Fem-PrEP study are underpowered to evaluate PrEP efficacy, as very few individuals take the study drugs. TDF2 is based on too few individuals and observation time. Therefore, any reasoning about distinct adherence requirements in women based on these studies may be statistically unsupported. When the remaining studies are dichotomized for “drug undetected” (≤ 1 dose/week) vs. “drug detected” (≥ 1 dose/week), PrEP efficacy estimates are ~90% in women (Fig. 2F). Notably, the precise level of adherence in these studies is still unknown, but any model involving local drug pharmacokinetics in vaginal and colon tissue homogenates substantially underpredicted this level of clinical efficacy, even when adherence was complete (Fig. 4 & 5). Notably, from a physiological point of view, tissue homogenate represents averages over potentially very heterogeneous drug concentrations in tissue-resident cells. Most of the tissue-resident cells in these homogenates are not targeted by HIV and consequently may not be relevant for the infection process, explaining why this marker may not predict PrEP efficacy. From our simulations, the most consistent scenario is the one where intracellular drug concentrations (TFV-DP and FTC-TP) in systemic PBMCs predict oral prophylactic efficacy. Physiologically, this marker is plausible: (i) It contains a large proportion of relevant HIV target cells (CD4+ T-cells) and (ii) oral FTC/TDF reaches the exposure site through the systemic circulation. Hence, PBMCs, as well as exposure-site-resident HIV target cells, may encounter very similar pro-drug levels that can be taken up and converted into active intracellular moieties.

Consequently, when using drug pharmacokinetics in PBMCs, the corresponding adherence-efficacy gradients suggest that PrEP protection is substantial when taken at least twice a week and interestingly, it is identical to adherence profiles derived for MSM [26, 48] where PrEP on demand is currently recommended.

## DISCUSSION

FTC/TDF-based PrEP adherence-efficacy profile in women has been highly controversial, fueled by apparent discrepancy between clinical trials evaluating PrEP in this risk group [9, 14, 17, 19, 20, 49].

The goal of our research was threefold: Through in-depth analysis of available clinical data, combined with advanced computational modelling of FTC/TDF-based PrEP, we wanted assess (i) whether the apparent discrepancy between clinical trials has statistical foundation, (ii) to challenge various mechanisms that have been proposed in the context of PrEP efficacy in women using clinical data and (iii) to synthesize adherence requirements that provide sufficient PrEP protection in women.

With regards to the available clinical studies, our population-pharmacokinetic modelling indicated that individuals having undetectable TFV plasma drug levels (< 0.001μM) must have taken FTC/TDF less than once a week, if at all (Suppl. Fig. S2–3). Since TFV plasma levels have been reported in a random sub-set of the PrEP intervention arm, we were thus able to dichotomize the intervention arms into sub-cohorts ‘undetectable drug’ (≤ 1 dose per week), and ‘detectable drug’ (≥ 1 dose per week). Interestingly, an identical dichotomization was performed to analyze trials in MSM, concluding that FTC/TDF-based PrEP is ~90% efficient in individuals who take *some* product [26].

When we assumed negligible PrEP efficiency for ‘undetectable drug’ sub-cohort and simulated the corresponding trials, we derived incidences closely matching incidences in the respective placebo arms. While serving as an internal control for our analysis, this finding also indicates that the intervention arm of the distinct studies contains variable ‘placebo-like’ observation periods, being either individuals that were never protected, or not protected for a period of time, supporting earlier observations in MSM [26]. This careful analysis of the various study outcomes alone could explain the bulk of apparent discrepancy between clinical studies with FTC/TDF-based PrEP in women. In essence, this analysis also pointed out that studies that reported low average PrEP efficacies (VOICE, Fem-PrEP) contain little or no information about the efficacy of PrEP in individuals that take (some of) the product, Fig. 2F. The remaining studies (Partners-PrEP, HPTN084) consistently translate into high PrEP efficacy for those individuals that take (some of) the drugs.

The consequential question was: How frequent does FTC/TDF need to be taken? Here, various proposed mechanisms aiming to justify distinct adherence-efficacy profiles in women provide different answers that allow us to challenge them with the clinical data. More specifically, we tested hypotheses related to the *route of exposure*, *exposure-site pharmacokinetics* and *exposure-site drug potency*. Our observation was that if PBMC was the relevant matrix measuring *effect-site* drug concentrations, the other proposed mechanisms did not alter PrEP efficacy and PrEP efficacy remained high (median efficacy: 90%), if FTC/TDF was taken twice per week on average (Fig. 5A–D). In simulations where local tissues were considered as the matrix measuring *effect-site* drug concentrations, the maximum achievable efficacy of FTC/TDF in fully adherent individuals was between 50–80% depending on the combination of tested hypotheses (Fig.5E–H). Also, in these scenarios, FTC/TDF would insufficiently protect from vaginal receptive intercourse (RAI), even if taken daily (Fig.3E,G), mainly because both TFV-DP and FTC-TP concentrations in vaginal tissue homogenates are several fold lower, compared to PBMC (Suppl. Text S3). Since these predictions were inconsistent with clinical data, we find the often-referred mechanistic explanation that women have intrinsically different adherence requirements because of local drug levels [25] quite unlikely. Instead, drug levels in exposure-site tissue may either be uninformative regarding oral FTC/TDF PrEP, or exposure-site pharmacokinetic models are still incompletely understood and need to be aligned with more sophisticated experimental (e.g. ex vivo) data, including topically applied PrEP.

Interestingly, when we simulated the various clinical sub-cohorts dichotomized for detectable plasma TFV, it appeared much more likely that FTC/TDF efficacy was high (~90%), in favor of hypotheses, in which PBMCs represent a more suitable matrix to determine effect-site drug concentrations (Table 2).

Notably, both matrices (PBMC, tissue homogenate) have advantages and disadvantages: Drug concentrations in PBMCs have been shown to correlate with drug concentrations in relevant CD4+ cell populations [26]. Moreover, during oral dosing, the drugs reach the virus exposure site through the systemic circulation. Hence, PBMCs, as well as exposure-site-resident HIV target cells, may encounter very similar pro-drug levels that can be taken up and converted into active intracellular moieties. However, systemic PBMCs may ignore potential differences between systemic and local sites, which can be particularly relevant for topically applied PrEP, when the drugs enter from the direction of the exposure site, with little or negligibly *systemic* drug concentrations.

On the other hand, local tissue biopsies contain a homogenate of HIV target- and non-target cells, where the latter may far outweigh the former in quantity. For NRTIs like tenofovir and emtricitabine this can be critical, since all processes from the uptake of the parent drug (TDF and FTC) into cells and their intracellular conversion to the active moieties (TFV-DP and FTC-TP) depend on the cell-specific abundance of relevant transporters and kinases [50, 51]. Hence, drug concentrations in tissue homogenates may reflect an average over a very heterogeneous mix that may not correlate well with concentrations in target-cells at the site of exposure.

In contrast to analysis by Cottrell et al. [25], we found modest impact of dNTP:NRTI-TP concentration ratios on FTC/TDF potency (Supplementary Text S3). One potential reason for this discrepancy is that Cottrell’s drug effect equations lacks any mechanistic justification in enzyme kinetics, affecting all downstream analysis. In particular, drug response in Cottrell et al. [25] scales directly with the dNTP:NRTI-TP ratio. This association may be incorrect, particularly when the molecular process of substrate or inhibitor binding become saturated. Notably, the enzyme kinetics of NRTI-TP incorporation into nascent viral DNA have been very well studied in pre-steady state single nucleotide incorporation assays [52] and reproducible kinetic parameters for drug- and substrate incorporation have been deduced therein (summarized in [30], Supplementary Table SN2.1) that strongly indicate that binding saturation can occur. This suggests that Cottrells’s model linking effect-site drug- and nucleoside concentrations with efficacy does not generalize well, and may be incorrect.

Another recent publication used a ‘top-down’ approach to analyze data from the HPTN084 (females) vs. HPTN083 (males) study to assess potentially different adherence requirements [53]. Although the adherence-protection profile is not statistically different in male and female participants, the study proposes higher adherence requirements in women, mainly based on TFV-DP levels in dried blood spots (DBS), that were used as adherence benchmarks. Notably, the previously introduced adherence benchmarks were based on a cohort of individuals who regularly took a proportion of the drugs. This detail is relevant, because TFV-DP has an extremely long half-life (about 17 days) in DBS; about four-fold longer than in PBMCs [54, 55]. This means that TFV-DP is still detectable in DBS, when there are no longer protective levels in PBMCs. In combination with decreasing adherence behavior, which was frequently observed in women [56–58] and infrequent drug testing, it thus possible that the recent analysis overcalled infections into moderate adherence categories (2–3, 4–6 doses per week). While HPTN084 clearly shows that none of the infected individuals had perfect adherence (Supplementary Text S4), aforementioned arguments may indicate that the adherence-risk reduction profile could in fact be steeper than suggested by the authors [53]. In fact, when inspecting the original data from HPTN084, only 4 out of 36 infected individuals (individuals E6, E30, E31, E36) show some evidence of taking the product around the time of infection (Supplementary Text S4; i.e. TFV-DP in DBS ≥ 350fmol/punch, which corresponds to ~1 dose per week on average [55]), while a large proportion seemed to have taken the product before a clinical visit, but not otherwise (and hence likely not around the time of infection), as evidenced by detectable plasma TFV levels coinciding with DBS TFV-DP < 350fmol/punch. The effectiveness of FTC/TDF PrEP in women could therefore be related to specific adherence behavior in this risk group [56–58], rather than intrinsic differences.

In summary, our investigation of FTC/TDF-based PrEP efficacy in women highlight that apparent discrepancy between clinical studies is statistically unsupported and can largely be attributed to different proportions of non-PrEP covered periods within the respective intervention arms. When dichotomizing the clinical trials accordingly, mechanistic models that consider drug concentrations in PBMCs are most consistent with the clinical data and predict adherence-efficacy profiles that are highly similar to historical analysis of the IPREX study in MSM: When the intervention arm is dichotomized into ‘drug detected’ vs. ‘drug undetected’, efficacy is about 90% [26]. In MSM, estimated efficacy was 76% (CI:56–96) for two doses per week, 96%(CI:90–99) for four doses per week, and 99% (CI: 96–99) for seven doses per week (compare Fig. 5A). When dried blood spots were used as adherence markers, ≤ two doses per week were associated with ~50% efficacy, 2–3 doses with ~87% and >4 doses with 86–100% efficacy [48]. Thus, the most plausible model from our analysis suggests that adherence-efficacy profiles in cisgender women are highly similar to MSM.

## METHODS

### Pharmacokinetics of FTC/TDF.

The two components of FTC/TDF (tenofovir disoproxilfumerate, TDF and emtricitabine, FTC) are prodrugs. FTC is taken up by cells and tri-phosphorylated intracellularly where it acts as an analogue of deoxycytosine triphophate (dCTP). TDF is first metabolized to tenofovir (TFV) by first pass liver metabolism and then twice phosphorylated to form TFV-DP, a deoxyadenosine triphosphate (dATP) analogue.

To fully reflect the pharmacokinetics of the two drugs, we utilized the previously developed models by Burns et al. and Garrett et al. [34, 35]. In both models, the amount of (pro-)drug in the dosing compartment (D), the amount of circulating compound in the central (=blood plasma, *A*_1_) and the peripheral (*A*_2_) compartment, as well as the amount of pharmacologically active metabolite (TFV-DP and FTC-TP respectively) in the cellular compartment (*A*_3_; peripheral blood mononuclear cells, PBMC) is considered. We calculated in units of *μ*mole internally to avoid unit conversions. The following ordinary differential equations (ODEs) were used to describe the mass-flux between aforementioned compartments, in between two dosing events:

(1) $$\frac{d}{dt}D=-k_{a}\cdot D\;\text{(dosing)}$$

(2) $$\frac{d}{dt}A_{1}=k_{a}\cdot D-k_{12}\cdot A_{1}+k_{21}\cdot A_{2}-k_{e}\cdot A_{1}-f_{13}\left(A_{1}\right)+f_{31}\left(A_{3}\right)\;\text{(plasma)}$$

(3) $$\frac{d}{dt}A_{2}=k_{12}\cdot A_{1}-k_{21}\cdot A_{2}\;\text{(peripheral)}$$

(4) $$\frac{d}{dt}A_{3}=f_{13}\left(A_{1}\right)-f_{31}\left(A_{3}\right)-f_{30}\left(A_{3}\right)\;\text{(cell)}$$

The terms *k*_*a*_ and *k*_*e*_ (1/h) denote the absorption- and elimination rate constants respectively. The terms *k*_12_ and *k*_21_(1/h) are the influx- and outflux rate constants to/from the peripheral compartment respectively. For the emtricitabine model, we used $f_{13}=\frac{V_{\text{max}}\cdot A_{1}}{KM+A_{1}}$, *f*_30_ = 0 and *f*_31_ = *k*_31_ · *A*_3_, where *V*_*max*_ (*μ*mole/h) and *KM* (*μ*mole) denote the parameters for the non-linear cellular uptake and intracellular conversion of FTC to FTC-TP and *k*_31_ denotes the rate constant of intracellular de-phosphorylation of FTC-TP to FTC and its efflux into the circulation.

For the tenofovir model, we used *f*_31_ = *k*_13_ · *A*_1_, *f*_30_ = *k*_30_ · *A*_3_, and *f*_31_ = 0, where *k*_13_ and *k*_31_ denote the rate constants of uptake, phosphorylation vs. efflux and de-phosphorylation respectively.

Between dosing events, the systems of ODEs was numerically integrated using *scipy.integrate.solveivp* in Python. At a dosing event *τ*_dose_, the amount of the drug in the dosing compartment *D* was elevated using the dosed amount (*μ*mole). Concentrations of the respective plasma prodrug concentrations and intra-cellular metabolite concentrations were derived by dividing by the respective volumes, i.e. *C*_1_ = *A*_1_/*V*_1_ and *C*_3_ = *A*_3_/*V*_3_ = *I*.

Pharmacokinetic parameter values for 1000 virtual patients were sampled from the distributions described in [34, 35] and are given in Supplementary Data Files 1–2. In line with the literature, we assumed that the two drugs do not interact with regards to their pharmacokinetics.

Adherence profiles were simulated by randomly drawing dosing events with a probability that corresponds to the weekly average dosing frequency. TDF mono-prophylaxis was simulated by simply not dosing FTC the corresponding dosing events.

### Clinical trial simulation.

We simulated the different clinical trials evaluating PrEP efficacy in women using Monte-Carlo simulations (Gillespie simulations). We set up a simple stochastic model with two reactions that simulate infection and drop out in the respective trials:

$$R_{1,\text{trial}}:S\overset{r_{\text{inf}}}{\to }I;\;R_{2,\text{trial}}:S\overset{r_{\text{dr}-\text{out}}}{\to }\emptyset .$$

Where *S* (‘susceptibles’) is initialized with the number of individuals in the respective clinical trial arm, the rate *r*_*inf*_ is either set to the incidence rate in the placebo arm (for simulation of the placebo arm), or it is set to the incidence in the placebo arm, multiplied with $1-\hat{\varphi }$ (average PrEP efficacy) to simulate interventions. The drop-out rate *r*_*dr*−*out*_ is reciprocally related to the follow up time in the respective clinical trial arm, i.e. $\text{average}\;\text{follow}-\text{up}\;\text{time}\;\text{per}\;\text{person}=\frac{1}{r_{\text{dr-out}}+r_{\text{inf}}}$. The average follow-up time per person is calculated from the number of individuals and the total observation time in person-years. Derived parameters are depicted in Table 1 and Fig .2.

### Local drug concentrations.

We simulated dosing schedules and measurement time-points for 7 studies that report local TFV-DP or FTC-TP concentrations [36–42] using the pharmacokinetic models exemplified above. Based on the respective simulated concentrations in PBMCs, we computed local:PBMC concentrations at the study-specific measurement time-points, as illustrated in Supplementary Text S3.

For evaluating prophylactic efficacy, we then calculated drug concentrations within local (colon, vaginal) tissues by multiplying the concentrations in PBMC (C_3_) by the tissue- and drug specific *weighted average* local:PBMC drug concentration ratios derived in Supplementary Text S3.

The *weighted geometric average* local:PBMC drug concentration ratios were calculated as $\frac{\sum_{i}w_{i}\cdot \text{log}\left(r_{i}\right)}{\sum_{i}w_{i}}$, where *i* denotes a particular study evaluating the local concentrations for the considered drug in the specific tissue, the weight *w*_*i*_ considers the statistical error of that study with $w_{i}=\sqrt{N_{i}}$ and *N*_*i*_ being the sample size of study *i*. Sample sizes for each data point were either reported in the respective studies or set to 1 if unreported.

### Pharmacodynamics of Truvada.

The intracellular triphosphorylated moieties FTC-TP and TFV-DP are nucleotide reverse transcriptase inhibitors (NRTIs) that compete with endogenous nucleotides (dCTP and dATP respectively) for incorporation into nascent proviral DNA during the reverse transcription of the viral RNA genome. Once incorporated, reverse transcription is (temporarily) halted, because FTC-TP and TFV-DP lack the necessary chemical group to attach the next incoming nucleotide during reverse transcription. In a previous work [31], we evaluated the combinatorial effect of FTC-TP and TFV-DP by extending a model for the MMOA of NRTIs for various drug-drug interaction hypotheses. The refined model acknowledges the fact that the combination therapy appears to decrease dNTP pools *in vivo* [59], which would favor NRTI incorporation and results in synergistic inhibition [31], while other mechanisms of interaction were found negligible at clinically relevant drug concentrations.

In this work, to speed up computation, we pre-calculated the combinatorial effects *η*(*I*_1_*, I*_2_) using the MMOA model for a 100 × 100 log-spaced grid of drug concentrations ranging from 0.001 … 150*μ*M for each of the two nucleoside reverse transcriptase inhibitors *I*_1_*, I*_2_ During PK-PD simulations we then derived *η*(*I*_1_(*t*)*, I*_2_(*t*)) for any combination of drug concentrations *I*_1_(*t*) = *C*_3_,_*TFV*−*DP*_(*t*) and *I*_2_(*t*) = *C*_3,*FTC*−*TP*_(*t*), encountered at time *t* by interpolating on the pre-calculated grid (*scipy.interpolate.griddata* [60] in Python).

### Local dNTP concentrations.

To simulate altered dNTP concentrations in local tissues at the virus exposure site (vaginal, colo-rectal), dNTP concentrations for vaginal and rectal tissues were extracted from Cottrell et al., [25] (Figure 1 therein). The MMOA model [46] was then run with these altered dNTP concentrations to compute the combinatorial effects of TFV-DP and FTC-TP, as outlined above.

### HIV replication model and PK-PD link.

To estimate infection and infection prevention by PrEP, we used a previously developed model of HIV replication [27, 29, 61]. In brief, the model consists of free viruses (V), as well as early- and late infected T-cells (*T*_1_ and *T*_2_ respectively). The replication cycle is modelled by six reactions with propensities *α*_1–6_ (eqs. (5)-(10)) that model the following processes: free viruses (V) can be cleared by the immune system or by unsuccessful infection of T-cells (reaction *R*_1_). This process is altered by NRTIs (like TFV-DP and FTV-TP) [30, 46, 62, 63] in the sense that the drugs increase the probability of unsuccessful infection. Pre-integrated virus in early infected T-cells (*T*_1_) can be cleared (reaction *R*_2_), and late infected T-cells (*T*_2_) can be cleared in reaction *R*_3_. Free virus can also successfully infect T-cells to form early infected T-cells with reaction *R*_4_. This reaction, too, is altered by the presence of NRTIs [30, 46, 62, 63]. I.e., the drugs inhibit the process of cell infection by inhibiting reverse transcription of the virus genome [30, 46]. In case of successful infection (and reverse transcription), proviral DNA may get integrated into the host DNA to form late infected T-cells (reaction *R*_5_), which produce new viral progeny with reaction *R*_6_. Utilized parameters are found in [33] (Table 1 herein). For HIV replication, we have the following reaction stoichiometries and reaction propensities:

(5) $$R_{1}:V\to *\;a_{1}\left(I_{1},I_{2}\right)=\left(CL+\left[\frac{1}{\rho_{\text{rev},\emptyset }}-\left(1-\eta \left(I_{1},I_{2}\right)\right)\right]\cdot \beta \cdot T_{u}\right)\cdot V$$

(6) $$R_{2}:T_{1}\to *\;a_{2}=\left(\delta_{\text{PIC}}+\delta_{{\text{T}}_{1}}\right)\cdot T_{1}$$

(7) $$R_{3}:T_{2}\to *\;a_{3}=\delta_{{\text{T}}_{2}}\cdot T_{2}$$

(8) $$R_{4}:V\to T_{1}\;a_{4}\left(I_{1},I_{2}\right)=\left(1-\eta \left(I_{1},I_{2}\right)\right)\cdot \beta \cdot T_{u}\cdot V$$

(9) $$R_{5}:T_{1}\to T_{2}\;a_{5}=k\cdot T_{1}$$

(10) $$R_{6}:T_{2}\to V+T_{2}\;a_{6}=N_{\text{T}}\cdot T_{2}$$

### Infection prevention (prophylactic efficacy).

Having modeled the time dependent effects of FTC/TDF on viral replication, we can estimate prophylactic efficacy using a recently developed numerical method in a matter of seconds [33].

The prophylactic efficacy *φ* is herein defined as the relative reduction in infection probability for a prophylactic regimen *S*, compared to the infection probability in the absence of prophylaxis ⌀ after virus challenge *Y*_*t*_:

(11) $$\varphi \left(Y_{t},S\right)=1-\frac{P_{I}\left(Y_{t},S\right)}{P_{I}\left(Y_{t},\emptyset \right)}$$

where *P*_*I*_(*Y*_*t*_, *S*) and *P*_*I*_(*Y*_*t*_, ⌀) denote the infection probabilities in the presence and absence of a *prophylactic regimen* *S*, if a given viral exposure *Y*_*t*_ occurs at time *t*. Here, a *prophylactic regimen* refers to a pharmacokinetic profile that is a consequence of a history of drug dosing, as well as individual pharmacokinetic parameters (Supplementary Data Files 1–2). For the absence of prophylaxis, *P*_*I*_(*Y*_*t*_, ⌀), analytical solutions have been presented in [61]. In order to estimate the probability of infection (and prophylactic efficacy) it is mathematically more convenient to compute the extinction probability *P*_*E*_, which is its complement

(12) $$P_{I}\left(Y_{t},S\right)=1-P_{E}\left(Y_{t},S\right)$$

To compute the extinction probability for a certain regimen, we used the method developed in [33]:

(13) $$\frac{dP_{E}\left(Y_{t}=\hat{V}\right)}{dt}=a_{1}(t)\cdot \left[P_{E}\left(Y_{t}=\hat{V}\right)-1\right]+a_{4}(t)\cdot \left[P_{E}\left(Y_{t}=\hat{V}\right)-P_{E}\left(Y_{t}=\hat{T_{1}}\right)\right]\;\frac{dP_{E}\left(Y_{t}=\hat{T_{1}}\right)}{dt}=a_{2}\cdot \left[P_{E}\left(Y_{t}=\hat{T_{1}}\right)-1\right]+a_{5}\cdot \left[P_{E}\left(Y_{t}=\hat{T_{1}}\right)-P_{E}\left(Y_{t}=\hat{T_{2}}\right)\right]\;\frac{dP_{E}\left(Y_{t}=\hat{T_{2}}\right)}{dt}=a_{3}\cdot [\left[P_{E}\left(Y_{t}={\hat{T}}_{2}\right)-1\right]+a_{6}\cdot [P_{E}\left(Y_{t}=\hat{T_{2}}\right)-P_{E}\left(Y_{t}=\hat{T_{2}}\right)\cdot P_{E}\left(Y_{t}=\hat{V}\right)]$$

where the time-dependent reaction rates *a*_1_(*t*) and *a*_4_(*t*) are computed according to eqs. (5), (8). The system of ordinary differential equations eq. (13) is solved *backwards* using standard ODE solvers as outlined in [33].

#### Modeling heterosexual virus exposure.

We investigated two modes of heterosexual exposures: receptive anal intercourse (RAI) and receptive vaginal intercourse (RVI). In simulations that (i) consider *local tissue* drug- and dNTP concentrations, differences between RAI and RVI arise due to differences in drug concentration, as well as drug potency at the two sites (outlined in Suppl. Text S3). Moreover, (ii) the amount of virus being transmitted and translocated to a physical site that enables productive viral replication (the inoculum size *Y*_*t*0_) is different for the two types of exposures. In simulations, we consider higher inoculum sizes for RAI, since the physiological barrier that separates donor virus from acceptor target cell environments only constitutes a single layer of epithelial cells (compare Fig. 3).

We have previously developed exposure models for receptive anal intercourse RAI [30]. In this work, the number of transmitted viruses that translocate to a physical site that enables productive viral replication (the inoculum size *Y*_*t*0_) was drawn from a binomial distribution; *Y*_*t*0_ ~ В(VL, *r*_RAI_), where we drew the virus load in the donor VL from a log-normal distribution (details in [30], Supplementary Text S1 therein) and derived the ‘success rates’ *r*_RAI_, such that average infection rates for unprotected sexual intercourse ${\hat{P}}_{I}(\text{RAI},\emptyset )$ coincide with reported values [11, 64]. For a purely receptive vaginal intercourse, we parametrized *r*_RVI_ accordingly using average infection rates for unprotected vaginal exposure [64–66], Suppl. Text S2.

Moreover, the ratio of RAI among total sexual acts in heterosexual women is also investigated to compute the PrEP efficacy at the population level. Since the estimation of this ratio varies in different studies [45, 67, 68], we investigated different frequencies *π*_*RAI*_ in the range 1–5%. It has been previously reported that 40% of heterosexual transmissions in women can be attributed to receptive anal intercourse [44, 45]. Thus, using $0.4=\frac{\pi_{\text{RAI}}\cdot {\hat{P}}_{I}(\text{RAI},\emptyset )}{\pi_{\text{RAI}}\cdot {\hat{P}}_{I}(\text{RAI},\emptyset )+\left(1-\pi_{\text{RAI}}\right)\cdot {\hat{P}}_{I}(\text{RVI},\emptyset I)}$, we can estimate *π*_*RAI*_ ≈ 4%. I.e. in simulations that consider anal- and vaginal receptive intercourse, 4% of exposures were simulated as RAI.

### Supplementary Material

1

## Figures and Tables

**Table 1.** Summary of clinical trials evaluating FTC/TDF-based PrEP in women.

	Intervention arm								Placebo arm	
Study	All		Drug detected			Drug undetected				
	T_*Tot*_	Inf_*Tot*_	T	Inf_*obs*_	Inf_*clin_sim*_	T	Inf_*obs*_	Inf_*clin_sim*_	T	Incidence
HPTN 084	1951	36	1092 (1054,1132)	4	3 (0,17)	858 (819,897)	32	33 (19,49)	-	4.2 (2.5,6.3)
TDF2	359	7	287 (269,302)	2	3 (0,7)	72 (57,88)	4	4 (0,11)	355	6.2 (0.0,16)
Partners-PrEP	933	9	752 (728,774)	2,25	2 (0,7)	181 (159,205)	6,75	7 (2,16)	1024	4.2 (1.0,9.1)
VOICE	1092	61	317 (284,349)	8	8 (0,26)	755 (721,786)	53	53 (35,74)	1108	6.9 (4.5,9.6)
FEM-PrEP	702	33	261 (238,288)	8	8 (0,17)	451 (428,477)	25,8	25 (16,41)	706	5.8 (3.0,9.3)

The total observation time of women participating in the intervention arms of the respective trials (T_Tot_, in person-years) was dichotomized into “drug detected” vs “undetected” based on the fraction of samples with detectable plasma TFV. Likewise, the number of infections Inf_Tot_ were proportionally assigned based on the fraction of infected individuals with detectable plasma TFV (Inf_Obs_). We then performed clinical trial simulations of the ‘drug undetected’ sub-cohort (Methods and Supplementary Text S1), assuming 0% PrEP efficacy. Based on these simulations, the number of infections with ‘detectable drug’ was then calculated as Inf_Tot_ – Inf_clin_sim_(drug undetected), which allowed to estimate uncertainty in this quantity without having to make assumptions about the efficacy of PrEP (Methods and Supplementary Text S1). A placebo arm was not present in the HPTN084 study. Uncertainty in the placebo incidence was computed with corresponding clinical trial simulation (Methods and Supplementary Text S1).

**Table 2:** Number of infected individuals for different hypotheses and comparison with clinical trial data.

Scenario	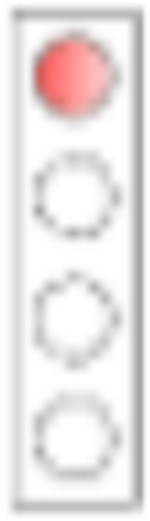	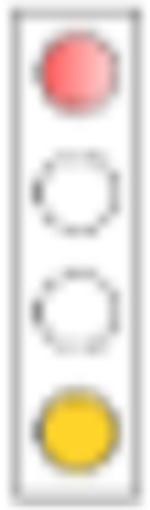	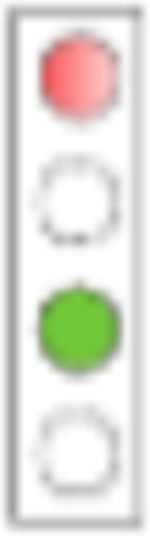	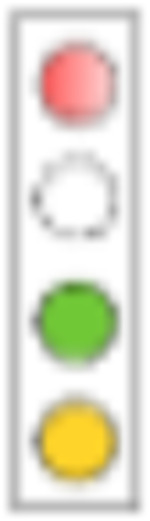	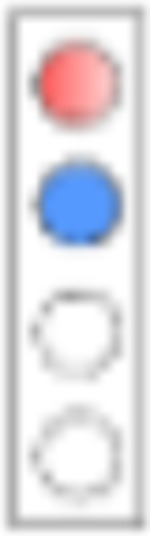	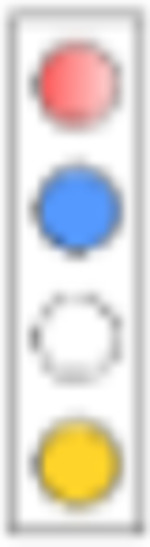	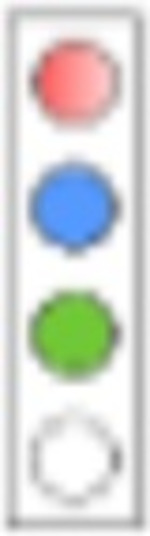	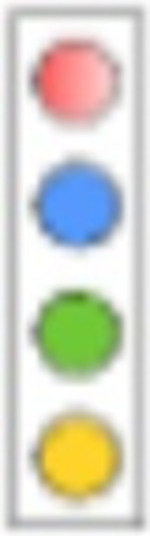	
PrEP efficacy (bottom-up)	91.1 (29.3, 99.9)	91.0 (28.9, 99.9)	93.4 (34.8, 100)	93.5 (37.0, 100)	35.8 (2.6, 60.4)	58.0 (12.4, 84.3)	48.2 (3.3, 71.5)	65.0 (15.7, 87.6)	
Inf_clin_sim_	**3 (0, 17)**								**HPTN 84**
Inf_sim_	4 (1, 9)	4 (1, 9)	3 (0, 7)	3 (0, 7)	27 (15, 42)	18 (9, 29)	22 (12, 35)	15 (7, 25)	
P-value	0.5	0.4996	0.5007	0.4911	0.0204 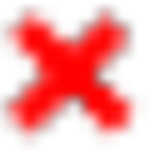	0.0903 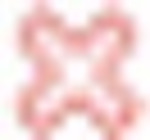	0.0483 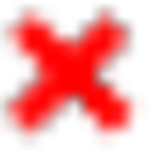	0.1399	
Inf_clin_sim_	**3 (0, 7)**								**TDF2**
Inf_sim_	1 (0, 5)	1 (0, 6)	1 (0, 4)	1 (0, 4)	11 (2, 27)	7 (1, 18)	9 (1, 22)	6 (1, 16)	
P-value	0.3986	0.3975	0.3487	0.3460	0.1412	0.2586	0.1936	0.3246	
Inf_clin_sim_	**2 (0, 7)**								**Partners-PreP**
Inf_sim_	3 (0, 8)	3 (0, 8)	2 (0, 6)	2 (0, 6)	20 (7, 41)	14 (4, 28)	16 (5, 34)	11 (3, 24)	
P-value	0.4412	0.4437	0.5106	0.5116	0.0204 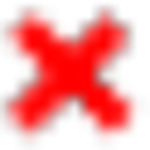	0.0606 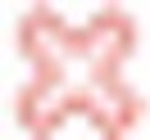	0.0357 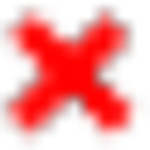	0.0972 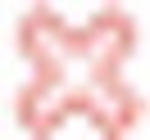	
Inf_clin_sim_	**8 (0, 26)**								**VOICE**
Inf_sim_	2 (0, 5)	2 (0, 6)	1 (0, 4)	1 (0, 4)	14 (7, 23)	9 (4, 17)	12 (5, 20)	8 (3, 15)	
P-value	0.3102	0.3123	0.2952	0.2881	0.2958	0.4448	0.3766	0.5039	
PrEP efficacy (bottom-up)	93.1 (38.8, 99.9)	93.0 (38.4, 99.9)	95.1 (45.4, 100)	95.2 (47.4, 100)	37.6 (3.9, 60.9)	60.0 (16.1, 84.6)	50.4 (5.1, 71.8)	67.1 (20.5, 87.8)	**FEM-PrEP**
Inf_clin_sim_	**8 (0, 17)**								
Inf_sim_	1 (0, 4)	1 (0, 4)	1 (0, 3)	1 (0, 3)	9 (3, 17)	6 (1, 12)	7 (2, 14)	5 (1, 10)	
P-value	0.2171	0.2193	0.2029	0.2039	0.4529	0.4435	0.5122	0.3976	

The first row depicts the distinct hypotheses in ‘traffic-light’ notation and the second row depict the ‘bottom-up’ estimated PrEP-efficacy in individuals with detectable plasma TFV, for that hypothesis (compare Fig. 5). The columns show the number of infected individuals and their respective confidence range, from ‘top-down’ clinical trial simulation Inf_clin_sim_ (compare Fig. 1) and from ‘bottom-up’ simulation Inf_sim_ with deduced PrEP efficacies for the distinct hypotheses. The *P*-value tests for differences in the number of infected individuals deduced from bottom-up modeling vs. clinical data. The *P-*value was empirically calculated by computing the proportion of 10^6^ simulation pairs, for which the null hypothesis was true (i.e. H_0_: *P* = number of simulations where infected individuals from hypothesis X was ≤ clinical estimate/total number of simulations, H_1_: number of infected individuals from hypothesis X > corresponding clinical estimate). Crosses visually indicate whether the statistical test provided trends (single unfilled red cross) or statistically different predictions at *P* < 0.05 (filled single- or double red cross).

## References

[1] Barre-Sinoussi F., , Isolation of a T-lymphotropic retrovirus from a patient at risk for acquired immune deficiency syndrome (AIDS). Science, 1983. 220(4599): p. 868–71. 6189183 10.1126/science.6189183

[2] Gallo R.C., , Isolation of human T-cell leukemia virus in acquired immune deficiency syndrome (AIDS). Science, 1983. 220(4599): p. 865–7. 6601823 10.1126/science.6601823

[3] Deeks S.G., , Research priorities for an HIV cure: International AIDS Society Global Scientific Strategy 2021. Nat Med, 2021. 27(12): p. 2085–2098. 34848888 10.1038/s41591-021-01590-5

[4] Roura M., , Barriers to sustaining antiretroviral treatment in Kisesa, Tanzania: a follow-up study to understand attrition from the antiretroviral program. AIDS Patient Care STDS, 2009. 23(3): p. 203–10. 19866538 10.1089/apc.2008.0129 PMC2776987

[5] Beattie T.S., , Personal, interpersonal and structural challenges to accessing HIV testing, treatment and care services among female sex workers, men who have sex with men and transgenders in Karnataka state, South India. J Epidemiol Community Health, 2012. 66 Suppl 2: p. ii42–48. 22495772 10.1136/jech-2011-200475

[6] Roberts D.A., , The impact of prevention-effective PrEP use on HIV incidence: a mathematical modelling study. J Int AIDS Soc, 2022. 25(11): p. e26034. 36385504 10.1002/jia2.26034 PMC9670193

[7] Murewanhema G., , HIV and adolescent girls and young women in sub-Saharan Africa: A call for expedited action to reduce new infections. IJID Reg, 2022. 5: p. 30–32. 36147901 10.1016/j.ijregi.2022.08.009 PMC9485902

[8] Mid-year population estimates 2021 (technical report). 2021, Department of statistics South Africa.

[9] Delany-Moretlwe S., , Cabotegravir for the prevention of HIV-1 in women: results from HPTN 084, a phase 3, randomised clinical trial. Lancet, 2022. 399(10337): p. 1779–1789. 35378077 10.1016/S0140-6736(22)00538-4 PMC9077443

[10] Landovitz R.J., , Cabotegravir for HIV Prevention in Cisgender Men and Transgender Women. N Engl J Med, 2021. 385(7): p. 595–608. 34379922 10.1056/NEJMoa2101016 PMC8448593

[11] Royce R.A., , Sexual transmission of HIV. N Engl J Med, 1997. 336(15): p. 1072–8. 9091805 10.1056/NEJM199704103361507

[12] Mayer K.H., , Emtricitabine and tenofovir alafenamide vs emtricitabine and tenofovir disoproxil fumarate for HIV pre-exposure prophylaxis (DISCOVER): primary results from a randomised, double-blind, multicentre, active-controlled, phase 3, non-inferiority trial. Lancet, 2020. 396(10246): p. 239–254. 32711800 10.1016/S0140-6736(20)31065-5 PMC9665936

[13] Hendrix C.W., HIV Antiretroviral Pre-Exposure Prophylaxis: Development Challenges and Pipeline Promise. Clin Pharmacol Ther, 2018. 104(6): p. 1082–1097. 30199098 10.1002/cpt.1227 PMC6925668

[14] Hodges-Mameletzis I., , Pre-Exposure Prophylaxis for HIV Prevention in Women: Current Status and Future Directions. Drugs, 2019. 79(12): p. 1263–1276. 31309457 10.1007/s40265-019-01143-8

[15] Sheth A.N., Rolle C.P., and Gandhi M., HIV pre-exposure prophylaxis for women. J Virus Erad, 2016. 2(3): p. 149–55. 27482454 10.1016/S2055-6640(20)30458-1 PMC4967966

[16] Baeten J.M., , Antiretroviral prophylaxis for HIV prevention in heterosexual men and women. N Engl J Med, 2012. 367(5): p. 399–410. 22784037 10.1056/NEJMoa1108524 PMC3770474

[17] Thigpen M.C., , Antiretroviral preexposure prophylaxis for heterosexual HIV transmission in Botswana. N Engl J Med, 2012. 367(5): p. 423–34. 22784038 10.1056/NEJMoa1110711

[18] Eshleman S.H., , Characterization of Human Immunodeficiency Virus (HIV) Infections in Women Who Received Injectable Cabotegravir or Tenofovir Disoproxil Fumarate/Emtricitabine for HIV Prevention: HPTN 084. J Infect Dis, 2022. 225(10): p. 1741–1749. 35301540 10.1093/infdis/jiab576 PMC9113509

[19] Van Damme L., , Preexposure prophylaxis for HIV infection among African women. N Engl J Med, 2012. 367(5): p. 411–22. 22784040 10.1056/NEJMoa1202614 PMC3687217

[20] Marrazzo J.M., , Tenofovir-based preexposure prophylaxis for HIV infection among African women. N Engl J Med, 2015. 372(6): p. 509–18. 25651245 10.1056/NEJMoa1402269 PMC4341965

[21] Kayesu I., , Uptake of and adherence to oral pre-exposure prophylaxis among adolescent girls and young women at high risk of HIV-infection in Kampala, Uganda: A qualitative study of experiences, facilitators and barriers. BMC Womens Health, 2022. 22(1): p. 440. 36357920 10.1186/s12905-022-02018-z PMC9648457

[22] Ngure K., , Dynamic Perceived HIV Risk and Sexual Behaviors Among Young Women Enrolled in a PrEP Trial in Kenya: A Qualitative Study. Front Reprod Health, 2021. 3: p. 637869. 36304002 10.3389/frph.2021.637869 PMC9580724

[23] Organization W.H., Differentiated and simplified pre-exposure prophylaxis for HIV prevention: update to WHO implementation guidance: technical brief. 2022, Geneva: World Health Organization.

[24] Molina J.M., , On-Demand Preexposure Prophylaxis in Men at High Risk for HIV1 Infection. N Engl J Med, 2015. 373(23): p. 2237–46. 26624850 10.1056/NEJMoa1506273

[25] Cottrell M.L., , A Translational Pharmacology Approach to Predicting Outcomes of Preexposure Prophylaxis Against HIV in Men and Women Using Tenofovir Disoproxil Fumarate With or Without Emtricitabine. J Infect Dis, 2016. 214(1): p. 55–64. 26917574 10.1093/infdis/jiw077 PMC4907409

[26] Anderson P.L., , Emtricitabine-tenofovir concentrations and pre-exposure prophylaxis efficacy in men who have sex with men. Sci Transl Med, 2012. 4(151): p. 151ra125. 10.1126/scitranslmed.3004006 PMC3721979 22972843

[27] Duwal S., , Hybrid stochastic framework predicts efficacy of prophylaxis against HIV: An example with different dolutegravir prophylaxis schemes. PLoS Comput Biol, 2018. 14(6): p. e1006155. 29902179 10.1371/journal.pcbi.1006155 PMC6001963

[28] Duwal S., Schutte C., and von Kleist M., Pharmacokinetics and pharmacodynamics of the reverse transcriptase inhibitor tenofovir and prophylactic efficacy against HIV-1 infection. PLoS One, 2012. 7(7): p. e40382. 22808148 10.1371/journal.pone.0040382 PMC3394807

[29] Duwal S., , The Utility of Efavirenz-based Prophylaxis Against HIV Infection. A Systems Pharmacological Analysis. Front Pharmacol, 2019. 10: p. 199. 30918485 10.3389/fphar.2019.00199 PMC6424904

[30] Duwal S., Sunkara V., and von Kleist M., Multiscale Systems-Pharmacology Pipeline to Assess the Prophylactic Efficacy of NRTIs Against HIV-1. CPT Pharmacometrics Syst Pharmacol, 2016. 5(7): p. 377–87. 27439573 10.1002/psp4.12095 PMC4961081

[31] Iannuzzi S. and von Kleist M., Mathematical Modelling of the Molecular Mechanisms of Interaction of Tenofovir with Emtricitabine against HIV. Viruses, 2021. 13(7). 10.3390/v13071354 PMC8310192 34372560

[32] von Kleist M., , HIV-1 polymerase inhibition by nucleoside analogs: cellular- and kinetic parameters of efficacy, susceptibility and resistance selection. PLoS Comput Biol, 2012. 8(1): p. e1002359. 22275860 10.1371/journal.pcbi.1002359 PMC3261923

[33] Zhang L., Wang J., and von Kleist M., Numerical approaches for the rapid analysis of prophylactic efficacy against HIV with arbitrary drug-dosing schemes. PLoS Comput Biol, 2021. 17(12): p. e1009295. 34941864 10.1371/journal.pcbi.1009295 PMC8741042

[34] Burns R.N., Hendrix C.W., and Chaturvedula A., Population pharmacokinetics of tenofovir and tenofovir-diphosphate in healthy women. J Clin Pharmacol, 2015. 55(6): p. 629–38. 25581815 10.1002/jcph.461 PMC5008110

[35] Garrett K.L., , A Pharmacokinetic/Pharmacodynamic Model to Predict Effective HIV Prophylaxis Dosing Strategies for People Who Inject Drugs. J Pharmacol Exp Ther, 2018. 367(2): p. 245–251. 30150483 10.1124/jpet.118.251009 PMC6170970

[36] Hendrix C.W., , Dose Frequency Ranging Pharmacokinetic Study of TenofovirEmtricitabine After Directly Observed Dosing in Healthy Volunteers to Establish Adherence Benchmarks (HPTN 066). AIDS Res Hum Retroviruses, 2016. 32(1): p. 32–43. 26414912 10.1089/aid.2015.0182 PMC4692123

[37] Hendrix C.W., , MTN-001: randomized pharmacokinetic cross-over study comparing tenofovir vaginal gel and oral tablets in vaginal tissue and other compartments. PLoS One, 2013. 8(1): p. e55013. 23383037 10.1371/journal.pone.0055013 PMC3559346

[38] Louissaint N.A., , Single dose pharmacokinetics of oral tenofovir in plasma, peripheral blood mononuclear cells, colonic tissue, and vaginal tissue. AIDS Res Hum Retroviruses, 2013. 29(11): p. 1443–50. 23600365 10.1089/aid.2013.0044 PMC3809387

[39] Ouattara L.A., , Genital Mucosal Drug Concentrations and anti-HIV Activity in Tenofovir-Based PrEP Products: Intravaginal Ring vs. Oral Administration. J Acquir Immune Defic Syndr, 2022. 89(1): p. 87–97. 34878438 10.1097/QAI.0000000000002820 PMC8647693

[40] Patterson K.B., , Penetration of tenofovir and emtricitabine in mucosal tissues: implications for prevention of HIV-1 transmission. Sci Transl Med, 2011. 3(112): p. 112re4. 10.1126/scitranslmed.3003174 PMC3483088 22158861

[41] Shieh E., , Transgender women on oral HIV pre-exposure prophylaxis have significantly lower tenofovir and emtricitabine concentrations when also taking oestrogen when compared to cisgender men. J Int AIDS Soc, 2019. 22(11): p. e25405. 31692269 10.1002/jia2.25405 PMC6832671

[42] Thurman A.R., , Safety and Pharmacokinetics of a Tenofovir Alafenamide Fumarate-Emtricitabine based Oral Antiretroviral Regimen for Prevention of HIV Acquisition in Women: A Randomized Controlled Trial. EClinicalMedicine, 2021. 36: p. 100893. 34041459 10.1016/j.eclinm.2021.100893 PMC8144741

[43] Bazzoli C., , Intracellular Pharmacokinetics of Antiretroviral Drugs in HIVInfected Patients, and their Correlation with Drug Action. Clin Pharmacokinet, 2010. 49(1): p. 17–45. 20000887 10.2165/11318110-000000000-00000

[44] Smith A.J. and Scott W.A., The influence of natural substrates and inhibitors on the nucleotide-dependent excision activity of HIV-1 reverse transcriptase in the infected cell. Curr Pharm Des, 2006. 12(15): p. 1827–41. 16724950 10.2174/138161206776873572

[45] O’Leary A., , Contribution of Anal Sex to HIV Prevalence Among Heterosexuals: A Modeling Analysis. AIDS Behav, 2017. 21(10): p. 2895–2903. 28058564 10.1007/s10461-016-1635-z

[46] Duwal S. and von Kleist M., Top-down and bottom-up modeling in system pharmacology to understand clinical efficacy: An example with NRTIs of HIV-1. Eur J Pharm Sci, 2016. 94: p. 72–83. 26796142 10.1016/j.ejps.2016.01.016

[47] Grievink H.W., , Comparison of Three Isolation Techniques for Human Peripheral Blood Mononuclear Cells: Cell Recovery and Viability, Population Composition, and Cell Functionality. Biopreserv Biobank, 2016. 14(5): p. 410–415. 27104742 10.1089/bio.2015.0104

[48] Grant R.M., , Uptake of pre-exposure prophylaxis, sexual practices, and HIV incidence in men and transgender women who have sex with men: a cohort study. Lancet Infect Dis, 2014. 14(9): p. 820–9. 25065857 10.1016/S1473-3099(14)70847-3 PMC6107918

[49] Celum C., , Acyclovir and transmission of HIV-1 from persons infected with HIV1 and HSV-2. N Engl J Med, 2010. 362(5): p. 427–39. 20089951 10.1056/NEJMoa0904849 PMC2838503

[50] Back D.J., , The pharmacology of antiretroviral nucleoside and nucleotide reverse transcriptase inhibitors: implications for once-daily dosing. J Acquir Immune Defic Syndr, 2005. 39 Suppl 1: p. S1–23, quiz S24–25. 15990598 10.1097/01.qai.0000168882.67942.3f

[51] Painter G.R., , Biochemical and mechanistic basis for the activity of nucleoside analogue inhibitors of HIV reverse transcriptase. Curr Top Med Chem, 2004. 4(10): p. 1035–44. 15193137 10.2174/1568026043388358

[52] Brown E.R., , Greater dapivirine release from the dapivirine vaginal ring is correlated with lower risk of HIV-1 acquisition: a secondary analysis from a randomized, placebo-controlled trial. J Int AIDS Soc, 2020. 23(11): p. e25634. 33206462 10.1002/jia2.25634 PMC7673220

[53] Anderson P.L., Marzinke M.A., and Glidden D.V., Updating the adherence-response for oral F-TDF for PrEP among cisgender women. Clin Infect Dis, 2023. 10.1093/cid/ciad021 PMC10209433 36645796

[54] Castillo-Mancilla J.R., , Tenofovir, emtricitabine, and tenofovir diphosphate in dried blood spots for determining recent and cumulative drug exposure. AIDS Res Hum Retroviruses, 2013. 29(2): p. 384–90. 22935078 10.1089/aid.2012.0089 PMC3552442

[55] Anderson P.L., , Intracellular Tenofovir-Diphosphate and EmtricitabineTriphosphate in Dried Blood Spots following Directly Observed Therapy. Antimicrob Agents Chemother, 2018. 62(1). 10.1128/AAC.01710-17 PMC5740314 29038282

[56] Murnane P.M., , Efficacy of preexposure prophylaxis for HIV-1 prevention among high-risk heterosexuals: subgroup analyses from a randomized trial. AIDS, 2013. 27(13): p. 2155–60. 24384592 10.1097/QAD.0b013e3283629037 PMC3882910

[57] Haberer J.E., , Understanding Pre-Exposure Prophylaxis Adherence in Young Women in Kenya. J Acquir Immune Defic Syndr, 2022. 89(3): p. 251–260. 35147580 10.1097/QAI.0000000000002876 PMC8826617

[58] Musinguzi N., , Trajectories of Oral PrEP Adherence Among Young Kenyan Women: Implications for Promoting Effective PrEP Use. AIDS Behav, 2022. 10.1007/s10461-022-03753-y 35841463

[59] Chen X., , Analysis of the Endogenous Deoxynucleoside Triphosphate Pool in HIV-Positive and -Negative Individuals Receiving Tenofovir-Emtricitabine. Antimicrob Agents Chemother, 2016. 60(9): p. 5387–92. 27353267 10.1128/AAC.01019-16 PMC4997838

[60] Virtanen P., , SciPy 1.0: fundamental algorithms for scientific computing in Python. Nat Methods, 2020. 17(3): p. 261–272. 32015543 10.1038/s41592-019-0686-2 PMC7056644

[61] Duwal S., , Mechanistic framework predicts drug-class specific utility of antiretrovirals for HIV prophylaxis. PLoS Comput Biol, 2019. 15(1): p. e1006740. 30699105 10.1371/journal.pcbi.1006740 PMC6370240

[62] von Kleist M., Menz S., and Huisinga W., Drug-class specific impact of antivirals on the reproductive capacity of HIV. PLoS Comput Biol, 2010. 6(3): p. e1000720. 20361047 10.1371/journal.pcbi.1000720 PMC2845651

[63] von Kleist M., , HIV quasispecies dynamics during pro-active treatment switching: impact on multi-drug resistance and resistance archiving in latent reservoirs. PLoS One, 2011. 6(3): p. e18204. 21455303 10.1371/journal.pone.0018204 PMC3063788

[64] Powers K.A., , Rethinking the heterosexual infectivity of HIV-1: a systematic review and meta-analysis. Lancet Infect Dis, 2008. 8(9): p. 553–63. 18684670 10.1016/S1473-3099(08)70156-7 PMC2744983

[65] Boily M.C., , Heterosexual risk of HIV-1 infection per sexual act: systematic review and meta-analysis of observational studies. Lancet Infect Dis, 2009. 9(2): p. 118–29. 19179227 10.1016/S1473-3099(09)70021-0 PMC4467783

[66] Leynaert B., Downs A.M., and de Vincenzi I., Heterosexual transmission of human immunodeficiency virus: variability of infectivity throughout the course of infection. European Study Group on Heterosexual Transmission of HIV. Am J Epidemiol, 1998. 148(1): p. 88–96. 9663408 10.1093/oxfordjournals.aje.a009564

[67] Elmes J., , Receptive anal sex contributes substantially to heterosexually acquired HIV infections among at-risk women in twenty US cities: Results from a modelling analysis. Am J Reprod Immunol, 2020. 84(2): p. e13263. 32384198 10.1111/aji.13263 PMC7485995

[68] Tian L.H., , Heterosexual anal sex activity in the year after an STD clinic visit. Sex Transm Dis, 2008. 35(11): p. 905–9. 18685549 10.1097/OLQ.0b013e318181294b

