# Supplementary material for: Synthesis of protective oral PrEP adherence levels in cisgender women using convergent clinical- and bottom-up modeling
Source: Res Sq. 2023 Apr 21:rs.3.rs-2772765. Preprint. [Version 1] doi: 10.21203/rs.3.rs-2772765/v1 (PMC10153398; doi:10.21203/rs.3.rs-2772765/v1)
Supplement: 1 [file NIHPPRS2772765V1-supplement-1.pdf]

Supplementary Figure S1: Population pharmacokinetic simulations of oral daily FTC/TDF. Population pharmacokinetic simulations for seven days oral 200/300mg dosing every 24hours in 1000 virtual patients. A & B: concentrations of the circulating prodrug (FTC, TFV), C & D: concentration of the intracellular active moiety (FTC-TP, TFV-DP) in PBMCs. The solid lines indicate the median, whereas the dark- and light grey areas present the quartile range and the 2.5% – 97.5% range respectively. The vertical dashed lines indicate the discontinuation of drug dosing after seven days. For visual guidance, the blue and yellow lines depict the fifty and ninety percent inhibitory concentrations (IC50, IC90), respectively.

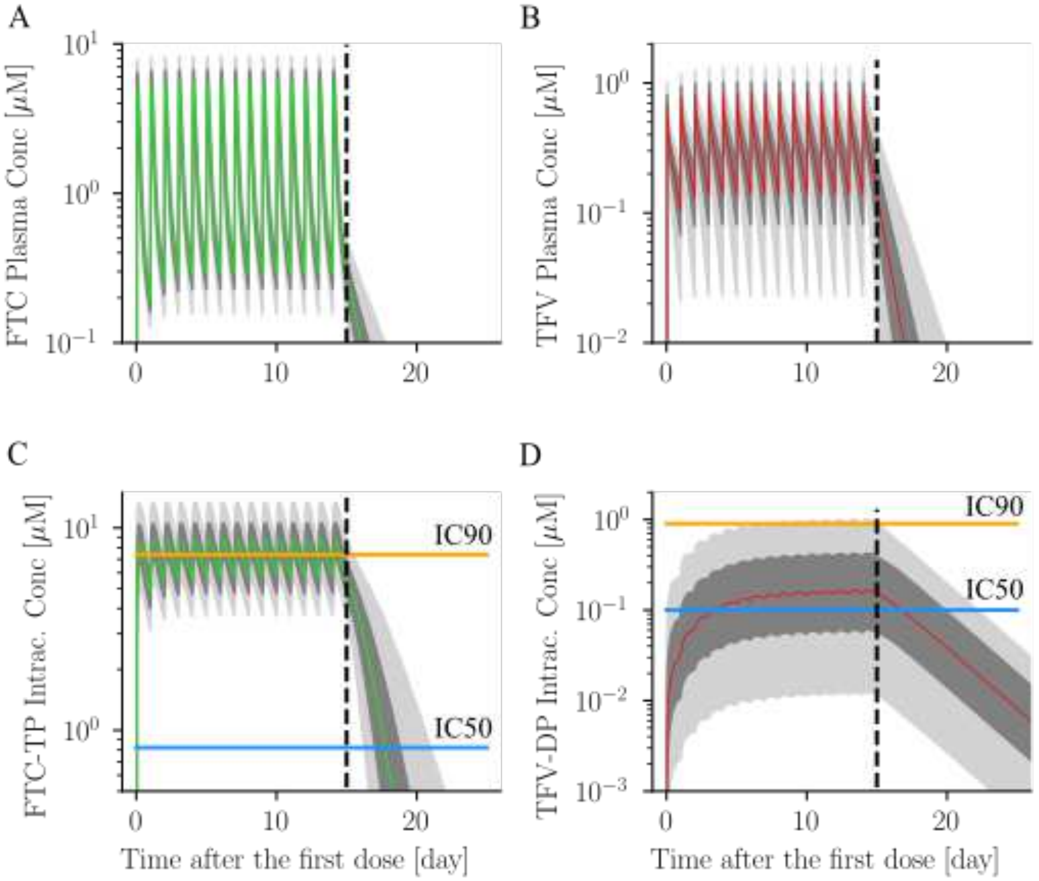

Supplementary Figure S2: Probability of detectable plasma TFV (LLOQ = 0.001µM) for different adherence levels. For each *average* adherence level, dosing profiles were randomly sampled. Plasma tenofovir (TFV) pharmacokinetis for oral 300mg TDF dosing were then simulated in 1000 virtual patients using our pharmacokinetic models (*Methods* section). For each adherence level, the percentage of time points where the plasma TFV was above the lower limit of quantification (LLOQ = 0.001µM) were then calculated. The solid lines indicate the median, whereas the dark- and light grey areas present the quartile range and the 2.5% – 97.5% range respectively. The vertical dashed lines indicate the adherence level with one-, two-, …, seven doses per week *on average*. The numbers above the plot represent the number of doses per week and the probability that plasma TFV is detectable. LLOQ = 0.001µM is related to clinical studies HPTN 084, Partners-PrEP, TDF2 and VOICE [6, 13, 14, 17].

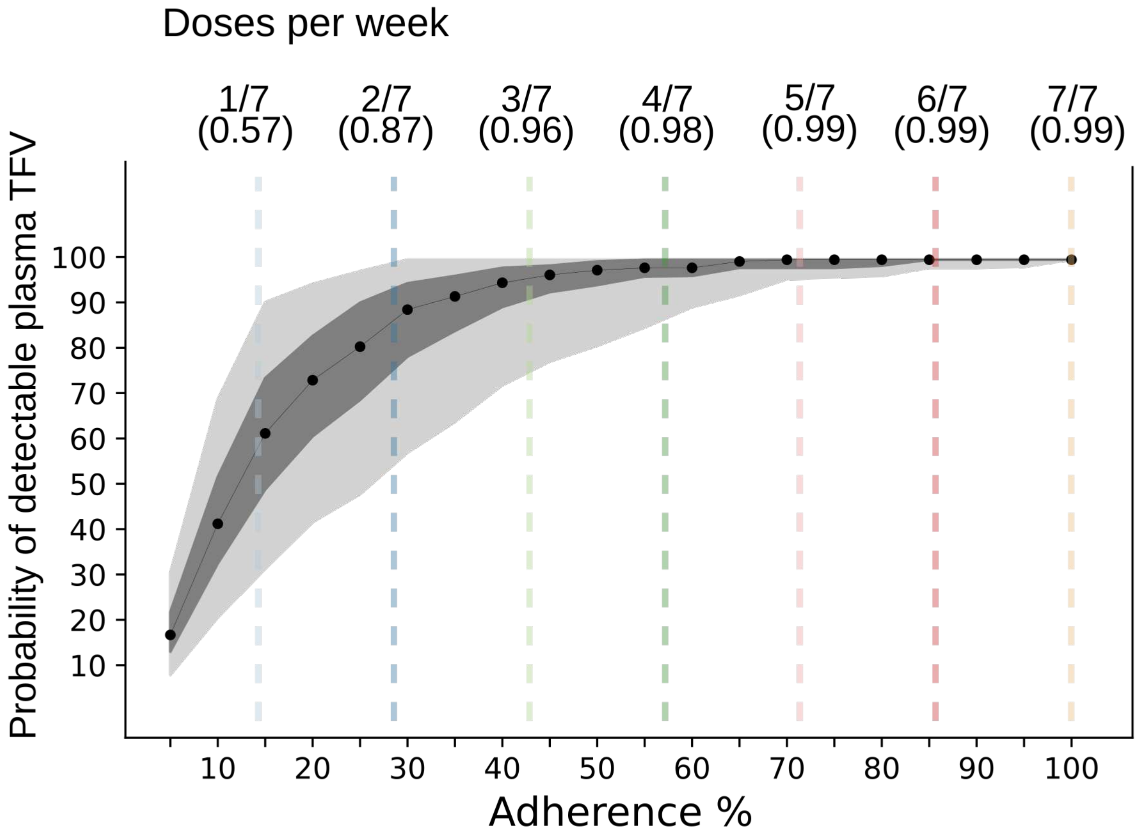

Supplementary Figure S3: Probability of detectable plasma TFV (LLOQ = 0.035µM) for different adherence levels. For each *average* adherence level, dosing profiles were randomly sampled. Plasma tenofovir (TFV) pharmacokinetis for oral 300mg TDF dosing were then simulated in 1000 virtual patients using our pharmacokinetic models (*Methods* section). For each adherence level, the percentage of time points where the plasma TFV was above the lower limit of quantification (LLOQ = 0.035µM) were then calculated. The solid lines indicate the median, whereas the dark- and light grey areas present the quartile range and the 2.5% – 97.5% range respectively. The vertical dashed lines indicate the adherence level with one-, two-, …, seven doses per week *on average*. The numbers above the plot represent the number of doses per week and the probability that plasma TFV is detectable. LLOQ = 0.035µM is related to the FEM-PrEP study [16].

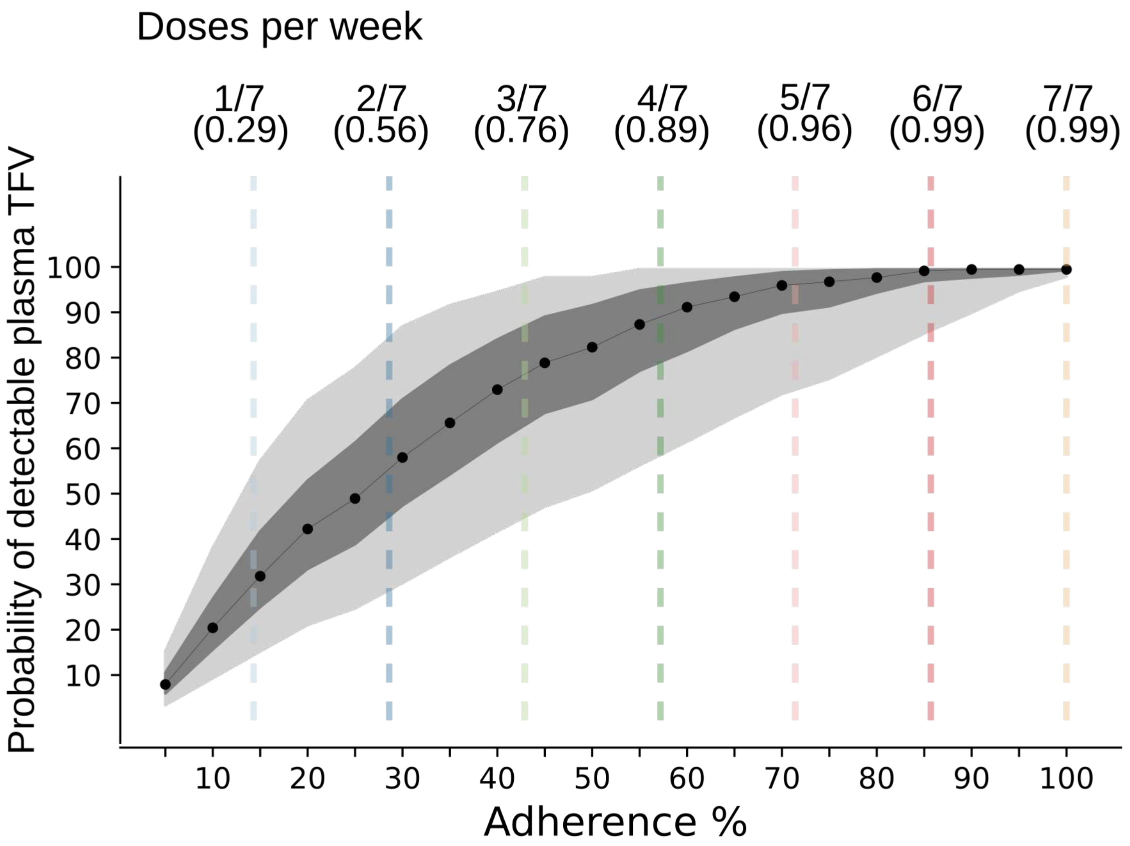

Supplementary Figure S4: Incidence in placebo and 'drug-undetected' sub-cohort of the PrEP intervention arms. Incidence rates in the placebo arms (blue error bars) and in the sub-cohort of the intervention arm where individuals had undetectable plasma TFV (orange error bars). Error bars show the mean incidence rates and the 95% confidence intervals computed using Wilson's method. Purple error bars show incidences (and their 95% confidence interval) computed from stochastic clinical trial simulations (*Methods*) with sampled incidence rates (Supplementary Text S1). Consequently, purple error bars depict uncertainty in the incidence rate, as well as intrinsic randomness in trial outcomes due to rare events, while the blue- and orange error bars only depict uncertainty in the incidence estimation due to sample size and the duration of observation.

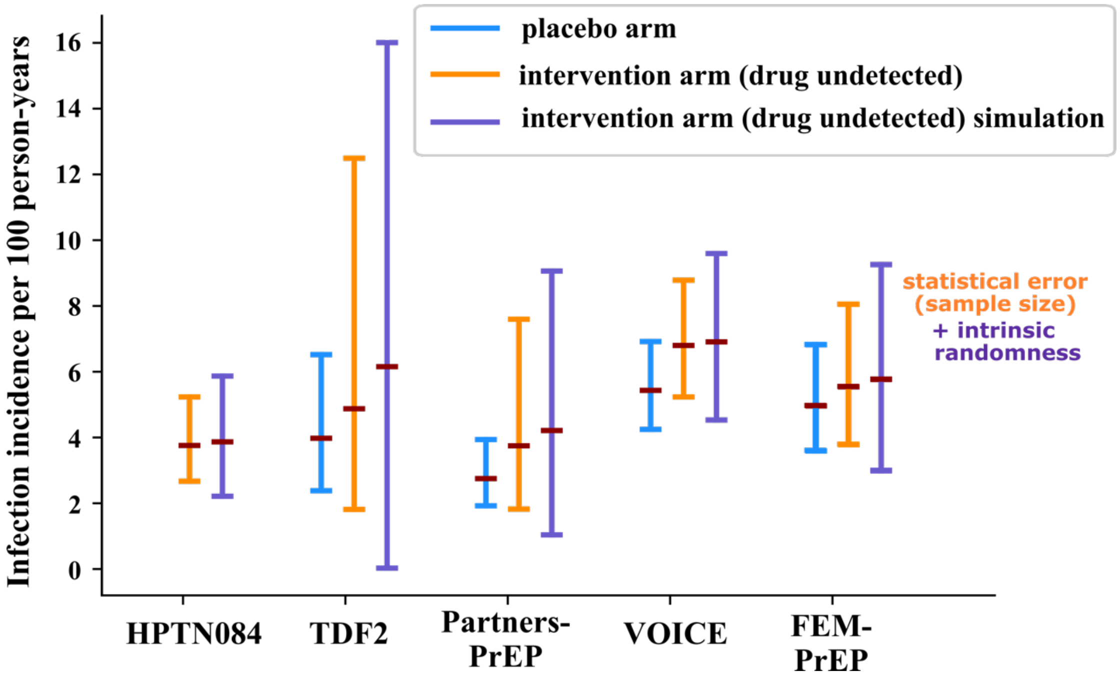

Supplementary Figure S5: Combined effect of FTC-TP and TFV-DP. The combination effect at clinically relevant concentrations of FTC-TP (concentration range: 0.03-1.44μM) and TFV-DP (concentration range: 10-66.66μM) was computed from a molecular mechanisms of action model (MMOA, *Methods* section). In the MMOA model, FTC-TP and TFV-DP molecularly interact by depleting the concentrations of endogenous deoxynucleotide levels, leading to a synergistic inhibition of reverse transcription. Computed values from the MMOA model have been interpolated to allow rapid calculation of their combined effects during PrEP simulations.

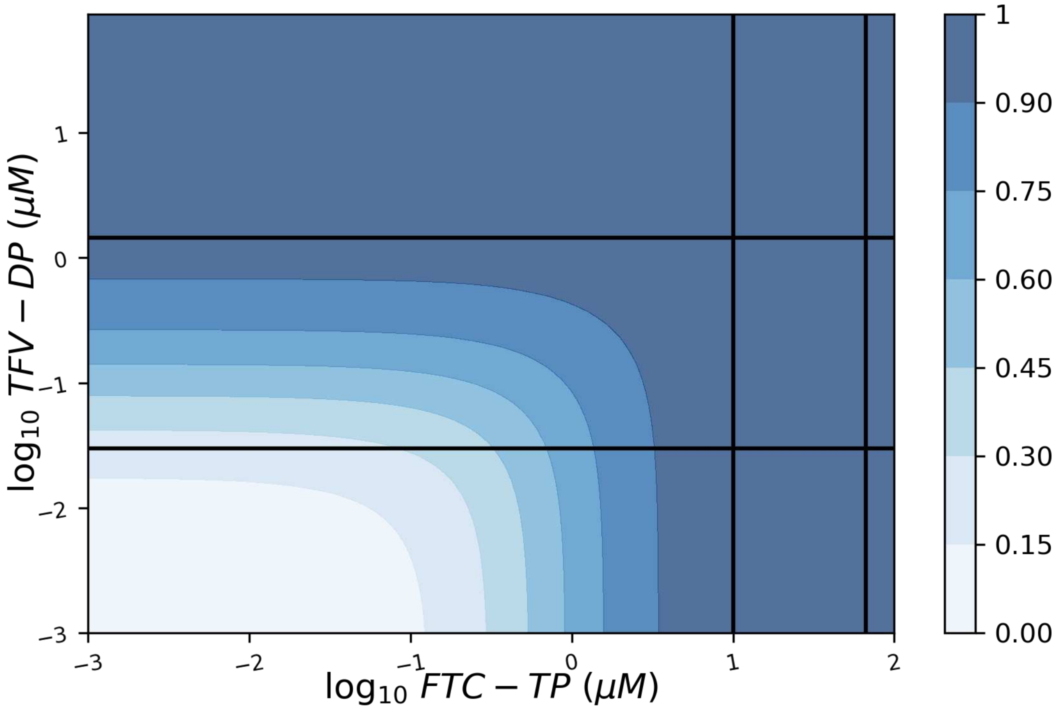

Supplementary Figure S6: Utilized adherence levels for bottom-up simulations in Table 2. Utilized adherence levels to compute PrEP efficacies for different 'bottom-up' modelling scenarios were computed based on the probability that TFV is detectable at a given adherence level. The probability of a certain adherence level in terms of j = 1…7 doses per week was computed as $P_j = \frac{P_j(TFV>LLOQ)}{\sum_{j=1}^{7} P_j(TFV>LLOQ)}$, where P_j(TFV >LLOQ) denotes the average probability that TFV is detectable (above the lower limit of quantification; LLOQ) for j weekly doses, as depicted in Supplementary Figure S2 and S3. A: Utilized adherence levels for LLOQ = 0.001µM (HPTN 084, Partners-PrEP, TDF2 and VOICE study). B: Utilized adherence levels for LLOQ = 0.035µM (FEM-PrEP study).

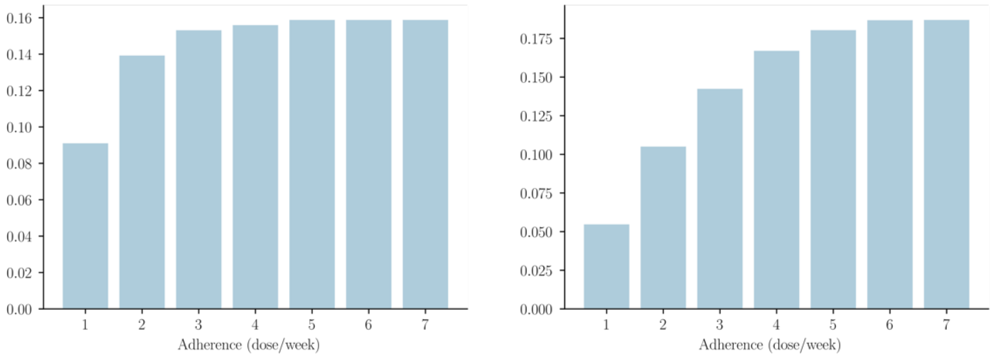

Supplementary Text S1: Mathematical details regarding the clinical trial simulation.

Supplementary Text S2: Details regarding the proportions- and the modelling of virus exposure routes in women through receptive vaginal- and receptive anal intercourse (RVI and RAI).

Supplementary Text S3: Details regarding the inference of drug levels in exposure-site tissue and exposure-site drug potency.

Supplementary Text S4: In-depth analysis of infection events in HPTN 084.

Supplementary Data File 1: Pharmacokinetic parameter values used in the FTC/FTC-TP model: KA, K12, K21, Ke, K31 in units 1/hours, Vmax and KM in units μM/h and μM respectively. The central and cellular compartment volumes (V1 and V3) were expressed in Liters and used for unit conversion (μmol to μM) after computation of the pharmacokinetic trajectory.

Supplementary Data File 2: Pharmacokinetic parameter values used in the TFV/TFV-DP model: KA, K12, K21, Ke, K13 and K30 are in units 1/hours. The central compartment volume V1 is expressed in Liters and was used for unit conversion (μmol to μM) after computation of the pharmacokinetic trajectory.
